# MADCAP: isolation of novel nAb-naïve AAV capsids from metagenomic data

**DOI:** 10.1128/jvi.00630-26

**Published:** 2026-09-09

**Authors:** Eugenia Lyashenko, Tess Torregrosa, Alexandra B. Ysasi, Jason Wu, Jie Bu, Margaret Hennessy, Youngji Na, Michael J. Ryan, Mehmet Takar, Rachna Manek, Joshua A. Hull, Edith L. Pfister, Christian Mueller, Sourav R. Choudhury

**Affiliations:** 1 Genomic Medicine Unit, Sanofi 201256 , Waltham, Massachusetts, USA; Cornell University Baker Institute for Animal Health, Ithaca, New York, USA

**Keywords:** gene therapy, evolution, parvovirus, AAV, metagenomic mining

## Abstract

Gene therapy using adeno-associated virus (AAV) vectors offers promising treatment for genetic disorders, but significant limitations restrict clinical application. Current AAV serotypes exhibit strong liver tropism and require high doses for extra-hepatic targeting, and pre-existing antibodies (NAbs) exclude up to 50% of potential patients. Evolutionarily distant isolates can evade neutralization but typically transduce human tissues poorly and require extensive engineering. We developed MADCAP (Metagenomic AAV Discovery and Capsid Annotation Pipeline) to systematically mine metagenomic data for functional, clinically relevant AAV capsids. We hypothesized that these sources might contain capsids that do not circulate widely in humans, can transduce human cells, and avoid neutralization. We screened 4.2 million metagenomic samples and identified 139 novel AAV capsid isolates which were tested for viral capsid assembly, viability, neutralization evasion, and tissue transduction in non-human primates. While natural serotypes (AAV1, AAV2, AAV9) were neutralized at low dilutions of pooled human immunoglobulin (IVIG), 68% of tested MADCAP capsids exhibited minimal to undetectable neutralization even at supra-physiological IVIG concentrations. Systemically delivered MADCAP capsids effectively transduced multiple clinically relevant tissues in non-human primates. Two capsids, MC46 and MC55, demonstrated improved CNS tropism compared to AAV9 while maintaining comparable production yields. In passive transfer studies, MC46 retained full transduction efficiency in the presence of human antibodies, while AAV9 transduction was completely lost. This work establishes metagenomic mining as a powerful tool for accelerating AAV capsid discovery, identifying isolates with favorable tissue tropisms and resistance to broadly neutralizing antibodies.

**IMPORTANCE:**

This work provides proof of concept that potentially clinically relevant AAVs can be isolated from metagenomic data. Our findings lay the groundwork for accelerated discovery of AAV capsids which could potentially increase the accessibility and effectiveness of AAV gene therapy.

## INTRODUCTION

AAVs are members of the genus *Dependoparvovirus* in the *Parvoviridae* family. Dependoviruses are small, nonenveloped viruses whose single-stranded DNA genomes contain two genes, one encoding non-structural proteins which facilitate viral genome replication, and the other encoding structural viral proteins (VPs) that make up the viral capsid. The first AAVs were discovered in the mid-1960s as contaminants in adenoviral preparations (1). To date, there are roughly 160 known natural AAV variants (2–4). While AAVs have been found in multiple vertebrate species (including birds and bats) (5, 6), most clinically relevant natural variants have been isolated from human and non-human primate (NHP) sources (2, 7).

Gene therapy has the potential to treat patients with genetic disorders that traditionally have had few treatment options. Adeno-associated virus (AAV) vectors are one of the most widely used delivery vehicles used in gene therapy today due to their ease of vectorization, broad (but sparse) human organ biodistribution and relatively low host immune response. However, AAVs suffer from some limitations that restrict their scope of use as clinical vectors. Many of them are largely liver tropic, and targeting extra-hepatic tissues often requires high doses, which present a safety risk (8). Pre-existing neutralizing antibodies (NAbs) to the AAV capsid, very prevalent for almost all clinical capsids (9), can limit transduction, making gene therapy ineffective and preclude use in a significant fraction of the eligible patient population. Furthermore, administration of AAV gene therapy leads to the generation of new NAbs, prohibiting the possibility of re-administration. For these reasons, a great deal of effort has gone into the search for novel capsids (10–12), and multiple capsid engineering approaches have been developed which aim to improve upon the properties of natural isolates. For example, avian AAVs have been successfully engineered to be epigenetically compatible for human transduction and are completely immune evasive from human isolates (13). However, those efforts require more complex vector engineering and have relatively lower success of identifying successful isolates with useful pre-existing tissue targeting (13). Here, we aim to leverage sophisticated algorithms, increasing computational power, and the vast amounts of available metagenomic data to understand what currently un-surveilled wild-type virions are circulating, and how they relate to known isolates and inform AAV biology. Our goal is to increase the diversity of AAV capsids and to uncover capsids that are not widely neutralized in the human population and can transduce human cells without extensive engineering or optimization.

While metagenomic profiling has been used extensively to elucidate bacterial and microbial diversity, studies of the viral component have been hampered by the lack of a comprehensive database of human viruses, the relative scarcity of viral reads in metagenomic samples, and by the computational expense of mining large next-generation sequencing (NGS) data sets. The COVID-19 pandemic saw a rise in the collection and profiling of metagenomic data designed to detect emerging zoonotic viruses (14, 15) which was accompanied by the development of sophisticated virome-mining algorithms (16–18). These methods, which use common viral genome features and “virus hallmark genes” to identify novel viruses, have resulted in a rapid expansion of the catalog of known viruses (19–22). Indeed, wastewater samples have provided a rich source for such discovery efforts (23).

Here, we present a method for mining AAVs in the metagenome. Using this method, we discovered 139 previously undescribed AAV capsids. Interestingly, a subset of capsids showed low to no neutralization by human NAbs even in the presence of antibody binding. Furthermore, a number of these capsids showed broad biodistribution in NHPs in a pooled assay. We further individually characterized two of these capsids, MC46 and MC55, and found that they exhibit widespread transduction of the NHP brain following intravenous delivery. In short, metagenomic mining enabled us to greatly expand the number of known AAV capsids and reveals capsids with the potential to overcome some of the immunological barriers to AAV gene therapy.

## RESULTS

### Discovery of novel AAV capsids by metagenomic data mining

We reasoned that mining of metagenomic samples would allow us to cast a wide net, potentially yielding novel AAV sequences from a wide range of sources. However, given the vast number of known viral species and the paucity of viral genome reads in metagenomic data, we expected the number of reads originating specifically from the *Parvoviridae* family to be very low, rendering their discovery computationally expensive. Therefore, we developed MADCAP (Metagenomic AAV Discovery and Capsid Annotation Pipeline), a three-step pipeline to mine for AAV capsids in metagenomic samples (Fig. 1A). We began by screening for *Parvoviridae*-containing reads using Serratus or a bwa reference-based alignment method. This pre-screening step eliminates sequence read archives (SRAs) where *Parvoviridae*-containing reads are not detected, significantly reducing the computational power required for subsequent *de novo* contig assembly and viral read annotation which is performed with Cenote-Taker 2 in *full* mode. This mode takes a sequencing read archive (SRA) file, assembles *de novo* all contigs using megahit, and then annotates viral contigs (16, 19). Next, we filter out contigs with parvoviral reads with a length less than 1,000 bp. Finally, we take contigs containing a group XIII Phospholipase A_2_ (PLA_2_) domain or the VP3 core jelly roll motif of the AAV VP1 capsid ORF (pfam08398 and pfam00740, respectively) and annotated as adeno-associated viruses. These domains were chosen as highly conserved, essential capsid motifs with relatively slow genetic drift (24–26) which makes them relatively easy to identify in divergent virions found in sequencing reads. To select for full-length VP1 proteins, only ORFs which were putative full-length (>683 amino acid) VP protein sequences with start and stop codons were selected, and these contigs were tested for BLAST similarity to known AAV capsids. We consider capsids novel if there is at least one amino acid difference from any known capsid. To confirm our method, we tested the ability of Cenote-Taker 2 to detect the AAV2 capsid in a contig known to contain the wild-type AAV2 genome sequence from NCBI (Fig. S1).

**Fig 1:**
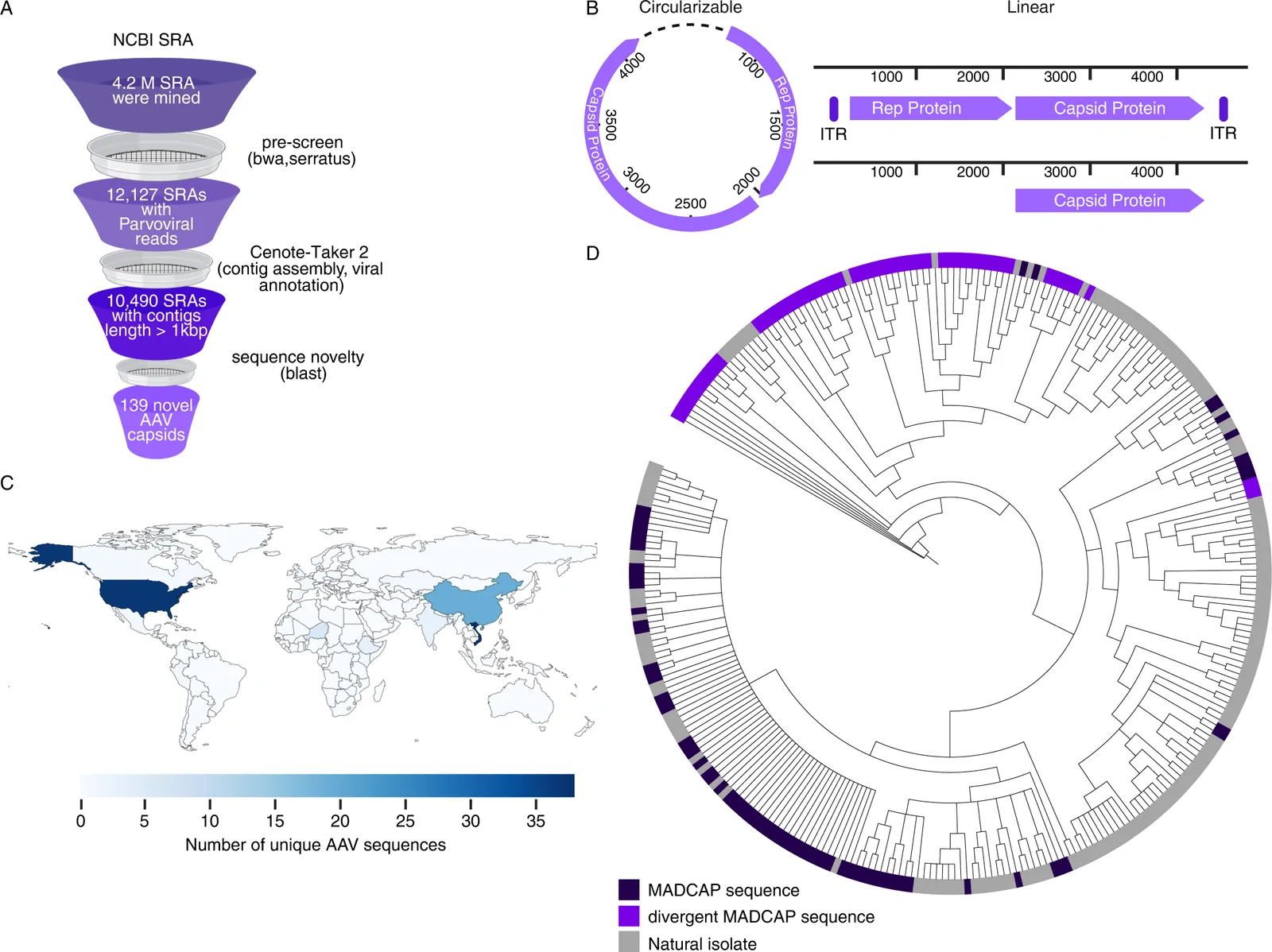
Discovery of novel AAV capsids by metagenomic data mining using MADCAP pipeline. (**A**) Schematic of MADCAP pipeline. (**B**) Representative architectures and features of viral AAV genomes discovered by MADCAP. (**C**) Global distribution of samples and novel AAVs found in them. The base map is from Natural Earth, with data added using GeoPandas. (**D**) Cladogram of known and novel AAV capsids. Displayed are branches with a bootstrap value of at least 50. MADCAP sequences are labeled divergent if they have less than 95% sequence identity to the natural isolates.

We mined 4.2 million SRA samples deposited over 16 years from around the world. We detected parvoviral reads in a total of 12,127 SRAs from 10,490 of which we assembled 2,201,180 contigs (Fig. 1A). Within complete contigs, i.e., those with end features (direct [16] or inverted [27] terminal repeats DTRs/ITRs), we found a variety of architectures (Fig. 1B) including circularizable and non-circularizable sequences. Due to the contig assembly process, reconstructed contigs within a given SRA may originate from multiple DNA/viral genomes rather than a single source. As such, it is possible they could represent a consensus of AAV (for example, in the quasi-species) within the SRA queried. For this reason, they must be validated as functional sequences.

Our pipeline identified 139 novel AAV sequences (Table S1), which we refer to as MADCAP sequences (Fig. 1C). These novel capsids are found across a range of branches of the AAV cladogram (Fig. 1D; File S1) and include sequences that diverge from their nearest known AAVs by between 58.4% and 99.9%. Next, we performed multiple sequence alignment of 49 novel AAV2-like MADCAP sequences against AAV2 (Fig. S2; Table S2). Thirty-nine of the 49 analyzed MADCAP sequences were related to the heparan sulfate proteoglycan (HSPG) negative binding isolates first identified by Chen and colleagues in 2005 (26); only 4 were identified as being closest related to wild-type AAV2 and putatively HSPG positive (26, 28). As expected for AAV capsids that are phylogenetically related, amino acid differences cluster in variable regions and the disordered linker domains of VP1/2, with a small number of variants observed outside of those regions (26, 28) suggesting that these represent valid AAV capsid sequences. Four AAV3B-like isolates were identified (MC66, MC67, MC136, and MC137) all of which lacked the position 594 arginine required for HSPG binding (Fig. S3A), strongly implying circulating AAV3B-like isolates are HSPG negative like true type AAV2 (26, 28, 29). For AAV1- and AAV6-like virions, only three examples were found in our survey (MC97, MC98, and MC132). For MC97, the previously characterized position 531 lysine required for HSPG binding was identified, strongly implying that it is the only capsid that is HSPG binding positive (Fig. S3B) (26, 28, 29).

A majority of these isolates were detected in the metagenome of wastewater, and there is no validation they represent infectious clones as unique isolates may contain singleton sequencing errors that can prevent successful viral production, packaging, or infection (30). To validate that these isolates were producible clones, we generated barcoded plasmid libraries comprising 92 MADCAP sequences and natural isolates AAV1-10. These were then pooled and packaged together using AAV2 Rep and a low quantity of input ITR plasmids (500 copies/cell) to reduce cross-packing effects (31) as has been previously reported (31). Quantification of capsid fitness by next-generation sequencing of DNase I resistant genomes showed successful packaging of a subset of MADCAP AAVs (Fig. S4A). These production yields span several logs suggesting that a subset of MADCAP capsids package too poorly for translation. A correlation plot of 10 packaging runs (Fig. S4B) confirms that packaging is reproducible. To confirm viral assembly, a subset of individually packaged MADCAP isolates was sent for negative stain transmission electron microscope imaging (Fig. S5), and the expected 26 nm particles consistent with past observations of empty and full capsids were identified. Taken together, these observations are consistent with MADCAP sequences representing natural assembly and packaging competent AAVs.

### Evolutionarily divergent novel MADCAP AAVs lack pre-existing neutralizing antibodies in humans

Within the general human population, AAVs circulate widely leaving hosts seroconverted with NAbs. Therefore, evasion of neutralizing responses can serve as a proxy for how widely circulating a given AAV isolate is within the human population. The high prevalence of NAbs against some AAVs in the general population presents a significant challenge to AAV gene therapy (9). The prevalence of NAbs to natural serotypes used in gene therapy applications is as high as 75% globally (9), substantially limiting patient access to gene therapies. We hypothesized that a smaller proportion of people might have NAbs toward more divergent capsids which do not widely circulate in the human population.

Based on divergence and packaging efficiency, we selected a subset of 41 capsids to perform NAb assays using human IVIG (purified immunoglobulin pooled from thousands of donors), which represents the pre-existing NAb pool in the general population. More than two-thirds (28/41 or 68.3%) of tested AAVs showed low or non-detectable neutralization to human pre-existing NAbs, suggesting that a subset of MADCAP capsids are capable of infecting human cells but do not circulate widely in the human population or cross-react with other circulating human isolates. We confirmed that this was not a function of using pooled IVIG by performing neutralization assays in 15 individual healthy donors (see Fig. 3D). This implies there is a large, untapped reservoir of potentially human-infecting dependoparvoviral virions, which could potentially dramatically expand the pool of patients eligible for gene therapy. We measured transgene expression following incubation with increasing concentrations of IVIG and compared percent neutralization of 41 AAVs to the natural serotypes AAV1, AAV2, and AAV9 (Fig. 2A; Fig. S6). While the natural serotypes were neutralized at the lowest concentrations of IVIG (50% neutralization titers ≤ 1:64 or ≤3,125 µg/mL), 28 out of 41 of the MADCAP AAVs showed low to non-detectable neutralization even at supraphysiologic (12,500 µg/mL) IVIG and a 50% neutralization titer could not be established, demonstrating a robust and reproducible absence of neutralization. For the 13 remaining capsids, neutralization titers differed significantly from the natural serotypes at multiple IVIG dilutions (Fig. 2B; Fig. S7). Mapping these novel AAVs onto a cladogram reveals distinct clusters corresponding with neutralization activity including clusters exhibiting undetectable neutralization (Fig. 2C).

**Fig 2:**
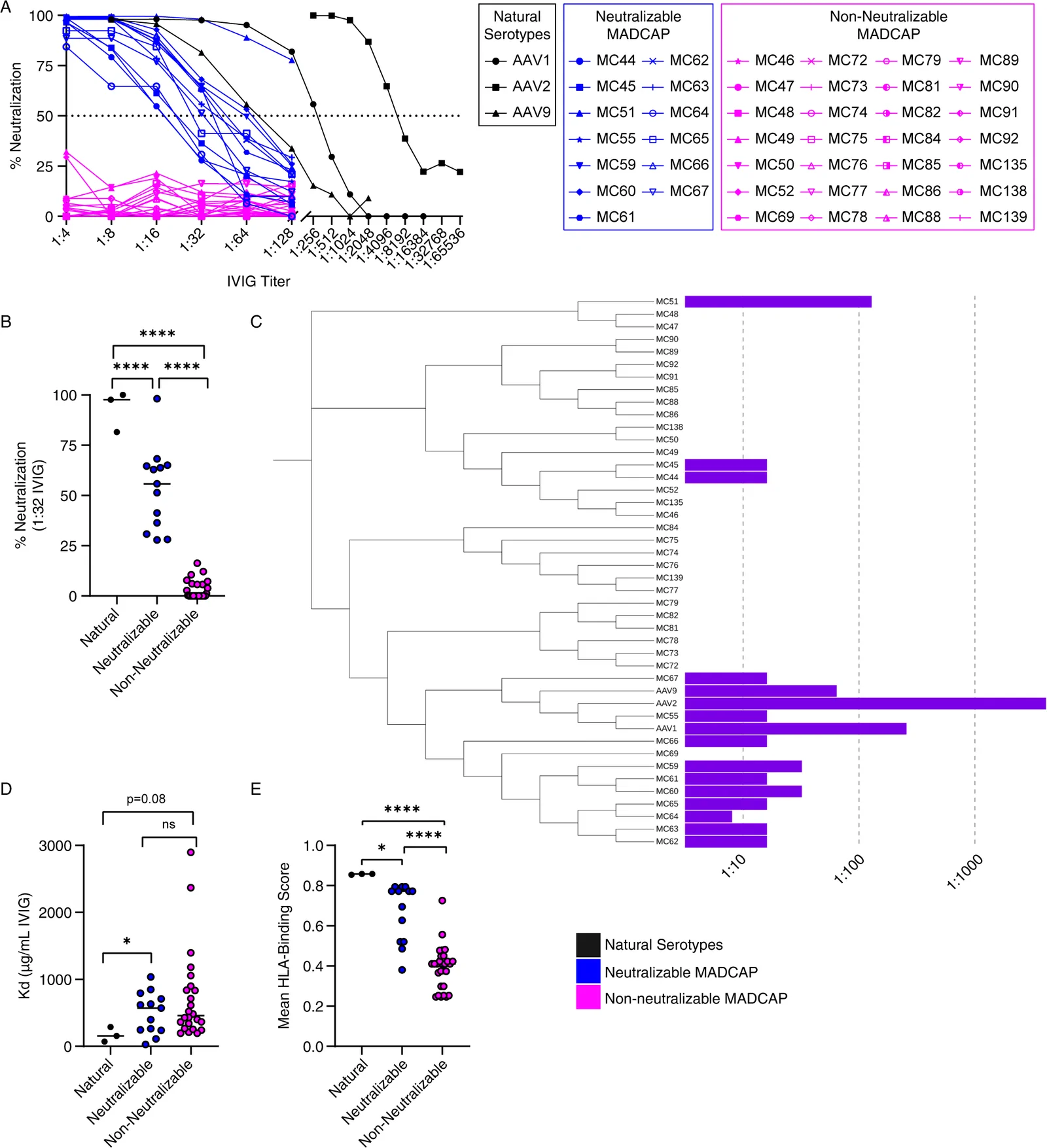
Evolutionarily divergent novel MADCAP AAVs lack pre-existing neutralizing antibodies in humans. (**A**) Individual percent neutralization curves for 41 novel clade MADCAP vectors along with natural isolate controls (black). (**B**) Percent neutralization at a 1:32 dilution of IVIG for natural serotypes (black) and MADCAP capsids grouped by neutralization status (neutralizable = blue; non-neutralizable = magenta). (**C**) Cladogram of MADCAP AAVs showing neutralization status. Bars indicate AAVs for which a 50% neutralization titer can be established. (**D**) Dissociation constants (Kd) for antibody binding for neutralizable (blue) and non-neutralizable (magenta) MADCAP capsids compared to natural serotypes (black). (**E**) *In silico* prediction of HLA-binding stability of known CD8 T-cell epitopes from AAV2, AAV9, and AAV6 for neutralizable, non-neutralizable, and known natural isolate AAVs. *P ≤ 0.05, ****P ≤ 0.0001.

All tested MADCAP capsids, including non-neutralized AAVs, bound total human antibodies in a concentration-dependent manner (Fig. S8). This binding interaction was sensitive to IgG-degrading enzyme of Streptococcus (IdeS) and, therefore, largely IgG-specific (Fig. S9). Natural serotypes, AAV1, AAV2, and AAV9, which are strongly neutralized, bound total antibody, to a larger extent, compared to both mildly neutralizable and non-neutralizable MADCAP capsids (Fig. 2D). This implies that structural divergence from those serotypes has led to overall lower antibody recognition. Intriguingly, the lack of significant difference in antibody binding between non-neutralizable and neutralizable MADCAP groups suggests that antibody binding alone does not determine neutralization status for these capsids (Fig. 2D). Indeed, while non-neutralizable capsids still bind to pre-existing human antibodies, binding does not interfere with cell transduction.

While less prevalent than pre-existing humoral immunity, pre-existing cytotoxic T lymphocyte (CTL)-mediated immunity is another major challenge in the AAV gene therapy field. CTL responses limit the expression of therapeutic transgenes and can induce cytotoxic tissue damage (32–35). We performed *in silico* predictions of processing and presentation of cytotoxic T-cell epitopes for MADCAP AAVs using publicly available tools (36). For peptide sequences that correspond to immunodominant epitopes in natural serotypes (mean HLA-binding score >0.85), both mildly neutralizable and, to a larger extent, non-neutralizable MADCAP AAVs (mean HLA-binding score <0.50) have significantly lower predicted HLA-binding scores, suggesting these epitopes have less stable binding to the HLA-I complex (Table S3; Fig. 2E). These capsid epitopes are, therefore, less likely to be presented to human T-cells, suggesting that some divergent MADCAP AAVs could escape cytotoxic T-cell responses inherent to natural serotypes.

### Some MADCAP AAVs broadly transduce non-human primate central nervous system

To further investigate their potential utility as gene transfer vectors, we explored the biodistribution and transduction profiles of MADCAP capsids in mice and in NHPs. C57BL/6J mice were treated with pooled AAVs intravenously at a dose of 2E12 vg/200 µL. To reduce the levels of neutralizing antibodies, NHPs were pretreated with IdeS (37) on day 0 and intravenous injection of the pool (5.54E13 vg/kg) was performed on day 3. In NHPs, several MADCAP AAVs exhibited the transduction of the CNS, muscle, and other peripheral organs (Fig. S10A and B). However, far fewer areas were transduced in mouse (Fig. S10C and D), and there was low correlation between the number of brain regions transduced in the two species with many of the MADCAP vectors transducing more brain regions in NHPs (Fig. S11). As the metagenomic data in these studies come primarily from human sources, this suggests that the MADCAP sequences could potentially represent early crossover events and have implications for translation, as it indicates that many MADCAP vectors would exhibit low transduction in rodent models.

An ideal gene therapy vector would have both negligible pre-existing neutralization and high specific transduction. Therefore, we focused on two capsids, MC46 and MC55, which exhibit pronounced CNS transduction relative to the clinical gold standard AAV9 and low (MC55) or no (MC46) neutralization by pre-existing B-cell immunity (Fig. 3A). MC46 and MC55 both displayed comprehensive brain penetrance, with superior gene transfer (DNA) and gene expression (RNA) to AAV9 in multiple brain lobes (occipital, parietal, and frontal), hippocampus, midbrain, thalamus, caudate, and pons (Fig. 3B), while retaining comparable transduction to AAV9 in primate liver. A single capsid study with seronegative NHPs would serve to validate and strengthen these biodistribution findings.

**Fig 3:**
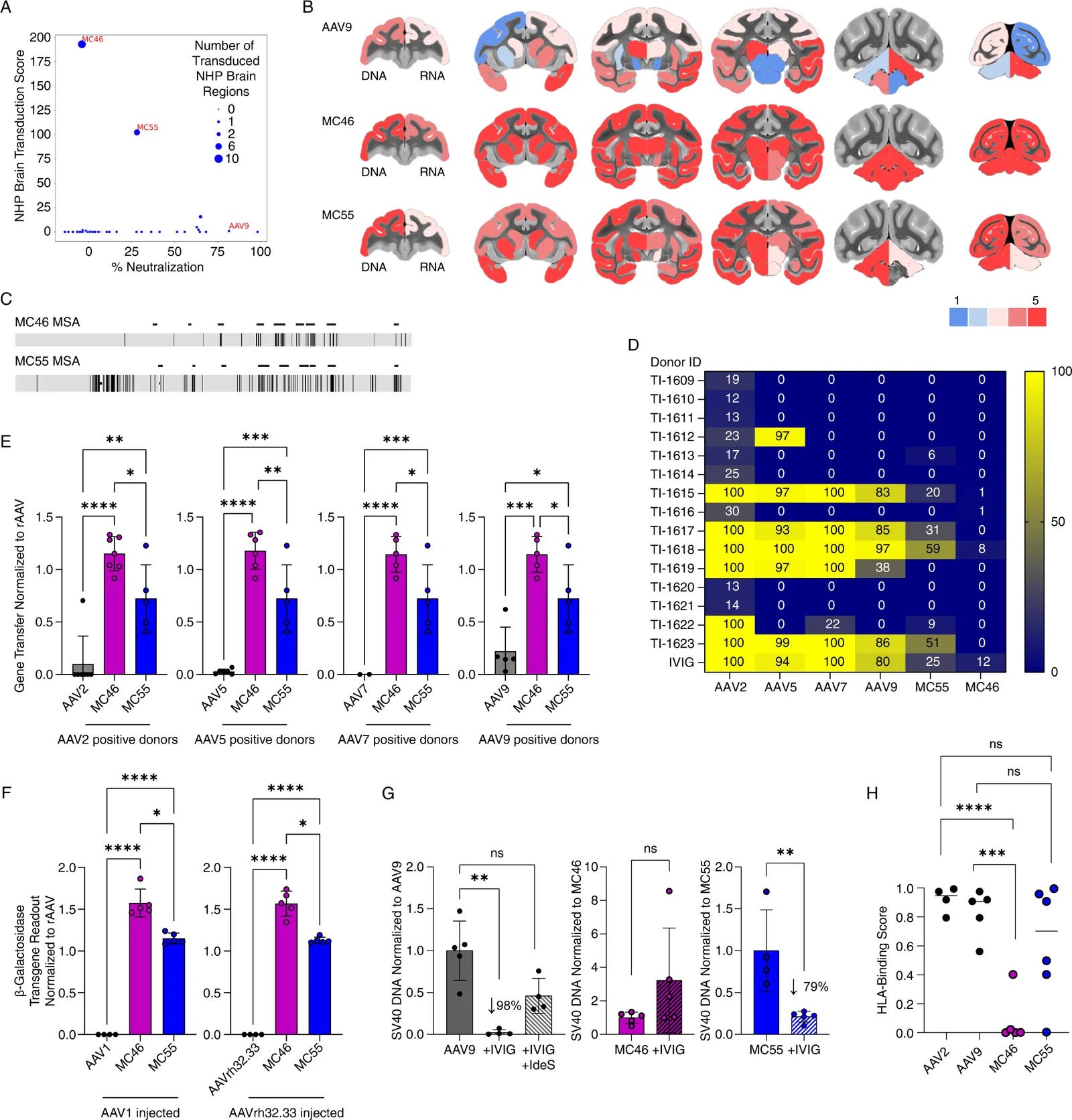
MADCAP AAVs show favorable biodistribution, transduction, and neutralization profiles in NHPs. (**A**) Neutralizable and non-neutralizable MADCAP AAVs MC46 and MC55 show high levels of brain transduction relative to AAV9 following IV injection in NHPs. (**B**) Biodistribution and transduction of AAV9, MC46, and MC55 in different brain regions of NHPs. (**C**) Sequence alignments of MC46 and MC55 show that mutations from AAV5 and AAV7, respectively, span the entirety of the MADCAP capsid sequence. (**D**) Percent neutralization for individual human donors natural serotypes. (**E**) Gene transfer of MC46 and MC55 in donors seropositive for natural serotypes. (**F**) Cell transduction efficiency in the presence of AAV1 and AAVrh32.33 capsid-specific NAbs from previously treated mice. (**G**) Transgene readout for biodistribution in mouse liver of MC46, MC55, and AAV9 with or without passive transfer of human IVIG. (**H**) *In silico* prediction of HLA-binding of known CD8 T-cell epitopes from AAV2, AAV9, MC55, and MC46. *P ≤ 0.05, **P ≤ 0.01, ***P ≤ 0.001, ****P ≤ 0.0001.

MC46 capsid is closely related to AAV5 (93.4% amino acid identity), while MC55 capsid is closely related to AAV7 (83.7% amino acid identity). Sequence alignments of MC46 and MC55 to AAV5 and AAV7, respectively (Fig. 3C), show that differentiated amino acids from these widely used gene therapy isolates are not only limited to variable regions but are spread throughout the entirety of the capsid sequence, suggesting that there might be important functional differences in conserved regions of the capsid.

To further validate the ability of MC55 and MC46 to evade pre-existing neutralizing antibodies, we quantified vector neutralization and gene transfer using serum from 15 healthy human donors. Natural serotypes AAV2, AAV5, AAV7, and AAV9 were strongly neutralized in at least five donors each, with the level of neutralization closely mirroring that of population-level screening using purified IVIG (Fig. 3D; Fig. S12). These same donors showed markedly decreased or absent neutralization of MC55 and no neutralization of MC46. Both MC55 and MC46 were able to effectively transduce cells and complete gene transfer in the presence of neutralizing antibodies against AAV2, AAV5, AAV7, and AAV9, with MC46 displaying higher relative gene transfer compared to MC55 (Fig. 3E; Fig. S12). Of note, there was limited neutralization of MC55 by donors seropositive for AAV7, the closely related natural serotype to MC55, and no neutralization of MC46 by donors seropositive for its closely related natural serotype AAV5, indicating sufficient structural divergence of MC55 and MC46 in antibody binding domains (Fig. 3C). These studies using healthy human donor serum corroborate neutralizing antibody screening using purified human IVIG and demonstrate effective human NAb evasion by MC55 and MC46 at both the population level and individual donor level. However, most seropositive donors displayed overlapping seropositivity for multiple natural serotypes, complicating interpretation of cross-reactive NAbs.

We next analyzed serum from actively immunized mice to quantify NAb cross-reactivity between single recombinant AAV vectors. Mice dosed with AAV1 or AAVrh32.33 display a strong NAb response 10–12 weeks after intramuscular dosing. These anti-AAV1 or anti-AAVrh32.33 antibodies were unable to neutralize MC55 and MC46, with MC55 and MC46 both effectively transducing cells in the presence of AAV1 and AAVrh32.33 capsid-specific NAbs (Fig. 3F). Of note, MC46 showed improved cell transduction and gene transfer in the highest serum concentration conditions over rAAV alone (Fig. S13). While MC46 has shown a lack of cross-reactive NAbs across species, it shows only a moderate decrease in antibody binding (IVIG Kd of 682 µg/mL compared to the natural serotype average IVIG Kd of 172 µg/mL). This is consistent with previous reports that non-neutralizing binding antibodies can improve AAV tissue transduction over AAV treatment alone (38).

Given the advantageous immunological profile of these two capsids, we sought to investigate if they could retain their biodistribution even in the presence of human pre-existing human antibodies. In an *in vivo* IVIG passive transfer study in mice, comparing the ability to transduce liver tissue in the presence of IVIG, AAV9 exhibited a 98% reduction of gene transfer in the presence of human antibodies (Fig. 3G; Fig. S14A). Gene transfer was partially restored by treatment with IdeS (Fig. 3G). In contrast, a smaller reduction (79%) was seen with MC55, and no reduction in biodistribution was observed for MC46 (Fig. 3G). This suggests that MC46 (and MC55 to a lesser extent) can be employed with no deleterious effect on biodistribution even in AAV9 seropositive organisms.

Neutralization assays show MC46 is completely non-neutralizable by pre-existing human antibodies, with no observable 50% neutralization titer (Fig. S14B). MC55 is mildly neutralizable by pre-existing human antibodies though, to a lesser extent, than natural serotype controls (50% neutralization titer 1:16 [3125 µg/mL] IVIG) (Fig. S14B). *In silico* predictions of HLA-I binding to capsid peptide sequences in MC55/MC46 suggest that of the six peptide sequences corresponding to natural serotype immunodominant T-cell epitopes, three regions in MC55, and all six regions in MC46 show a notable reduction in predicted HLA-binding and, therefore, projected reduced CD8 T-cell reactivity to MC46 and MC55 (Fig. 3H).

To further characterize these putative capsid monomers, Boltz-2(39) was used to determine the predicted protein fold of the VP1 monomeric unit. For both, the capsid VP1 folds appeared to consist of a VP3 C-terminal 8 β-strand jelly roll motif with accessory βA strand typical of dependoparvoviruses; with a VP1/2 disordered linker, an ordered PLA_2_ domain, and generally an ordered far N-terminal helix (Fig. S15A). Overall, with regard to the ordered domains, the most structural variability was noted in or proximal to the previously characterized variable regions (VRs) and DE/FG/HI loop of the VP3 capsid core domain (Fig. S15B); while minimal positional precision was found between the PLA_2_ and VP3 core which are connected by a flexible linker, and likewise for the far N-terminal helix. Compared to previously characterized capsids AAV5 and AAV7, variant positions in MC46 and MC55 were largely located on the capsid ex-lumenal surface as would be expected of a domain undergoing genetic drift to escape host immunity and evolving receptor usage (Fig. S15C), with minimal variants on the lumenal surface and PLA_2_ domain (not shown). It is likely these ex-lumenal surface variants which give MC46 and MC55 broad NAb evasion compared to their relatives AAV5 and AAV7, respectively. Among the broadly neutralizing human isolated monoclonal antibodies, four examples have been demonstrated, and they map to the viral 5-fold axis of symmetry (2 clones of 2), 2-fold axis of symmetry (1 clone of 16), and the 2/5-fold wall (only clone) (40). However, none of those clones were reactive to AAV5. Among the AAV5 antibodies that are known to date with published contact residues are ADK5a (non-neutralizing), ADK5b (neutralizing), 3C5 (non-neutralizing), and HL2476 (neutralizing). While all four are mouse monoclonal antibodies, it is interesting to note that point mutations on MC46 overlap the binding footprints in AAV5 for all of these antibodies (Fig. S16). Thus, point variants of the capsid surface do not appear to be distinguishing specifically between neutralizing and non-neutralizing antibodies, possibly hinting that antibody affinity, as opposed to just binding pose, may be highly influential in distinguishing binding and neutralizing antibodies (41, 42).

Notably, both MC46 and MC55 have extensive variation compared to AAV5 and AAV7 near the threefold as well as the twofold symmetry axis to which most (76%) identified human NAbs have been found (40), with a smaller but meaningful number of variants near the fivefold symmetry axis (Fig. S15C). It is well documented that AAV5 (a close relative to MC46) is far less immunogenic than other AAV serotypes in humans, thus, acquiring two- and threefold mutations is likely sufficient to enable immune evasion from clade-specific antibodies. By contrast, MC55 is closely related to AAV7, which belongs more closely to widely circulating human virions, and, thus, requires variants of the fivefold as well (which is observed). This implies that rapid evolution of the two- and threefold to escape the human immune system which is biased toward these features of the capsid surface is likely the norm for AAVs to escape local minima of clade-specific antibodies, but that escape from broadly neutralizing antibodies requires acquisition of more difficult-to-acquire capsid variants at the fivefold symmetry axis. Thus, humans and AAV have found themselves in an arms race where the dominant clade-specific viral epitope(s) are the ones undergoing the most dramatic surface changes and the fivefold serves as a critical region to de-target broadly neutralizing anti-AAV antibodies.

## DISCUSSION

Current clinical-stage AAV gene therapies rely on a limited set of AAV capsids and their derivatives, the majority of which have been isolated from human and non-human primate genomes. This limited set of capsids has proven useful, but there is clearly a need for additional capsids more aligned with clinical expectations. To expand this repertoire, we built our own state of the art tool by taking advantage of vast public SRA data and existing tools (Serratus and Cenote-Taker 2). In the process, we uncovered 139 new AAVs among which we identified capsids that can transduce multiple regions of the primate brain following systemic delivery and are not neutralized by pre-existing human antibodies.

Our analysis uncovered both circularizable and non-circularizable genomic sequences. Circularizable sequences could arise from genomes actively undergoing rolling circle replication, or vectors generated from that method with imperfect terminal resolution (therefore containing a portion of a neighboring genome packaged), or perfect DTRs of extended length. Those non-circularizable could arise from genomes generated by rolling hairpin replication method. This aligns broadly with the literature that both rolling circle and rolling hairpin replication occur in wild-type AAVs dependent upon co-infecting virus (27, 43, 44). Our method only mined genomes containing the putative capsid gene open reading frame; however, many vectors with genome truncations were observed. Such vectors may result from multiple sources such as incomplete sequencing depth (e.g., a portion of the Rep contig was missing but present in the virus), or defective particles produced in nature; however, which of these cannot easily be ascertained after the fact.

For the identified wild-type virions, a bias of virion discovery was observed. Of the human infecting isolates, no virions related to AAV4-like clade (e.g., AAV4/11/12) were identified. Furthermore, given the high diversity of avian-AAV isolates, they were relatively rare (7/139 or 5%) in our survey. However, 35% (49/139) of virions found were AAV2-like making AAV2-like viruses the most common in our survey. This is likely because these samples primarily consisted of sewage and other areas of human interest in contrast to prior surveys which found avian isolates directly through cloacal swabs. Typically, in those avian surveys, the researchers were looking for wild-type viruses which were likely flagged upstream of our annotation pipeline and were, therefore, not unique isolates. Thus, it appears that AAV2-like viruses are likely one of the most widespread dependoparvoviruses worldwide. It is not surprising then that AAV2 pre-existing antibodies are so common in the human population. Additionally, of these AAV2-like virions, approximately 80% (39/49) were Chen-like isolates, implying that the circulating true type isolates of AAV2 continue to remain HSPG negative. This trend was consistent with AAV3B-like and AAV1/6-like virions and reinforces the idea that AAV HSPG usage is generally not observed in wild circulating virions, strengthening the assertion that HSPG is probably a cell culture adaptation and detrimental to circulating viruses.

When considering the VP1 fold of these virions, we noted that variants tended to cluster within disordered domains and the VP3 core ex-lumenal surface. This is not surprising as accumulation of variants in regions such as capsid interior and the PLA_2_ domain are more likely to have neutral or detrimental impacts relative to the gains from potential receptor switching, or immune evasion from variants of the VP3 core domain ex-lumenal surface. Fold certainty was unsurprisingly highest in the jelly roll motif of the VP3 core domain, as it has been conserved in most single jelly roll virions, so there is ample training data for Boltz-2 to learn from. In the VP3 core domain, the regions near symmetry axis extending from the jelly roll motif between the β-strands (the loops and variable regions) with heavy interdigitation were the least certain, as Boltz-2 is likely not relying on true physics but instead learned hallucination of these regions of the capsid monomer to properly fold them, and they also tend to be ex-lumenal and thus the most variable. Some capsids had predicted helixes in the VP1/2 linker though with low confidence and reproducibility; this could be a model hallucination. Only a single capsid lacked the ordered far N-terminal helix (MC52); however, this vector appeared to have a larger ~50 aa deletion from the NTD and is likely therefore an incomplete virion sequence. While we repeatedly observed an additional far N-terminal helix, there is no strong evidence that this domain exists as an ordered structure though we noted that this helix tended to be positioned in the PLA_2_ active site. However, the region is likely essential for epigenetic maintenance of the episome (13); therefore, the 683 aa cutoff plausibly explains the recovery of MC52 is likely too generous and could be increased. The lack of structural variation predicted within the PLA_2_ domain corresponds with observations from previous surveys of dependoparvoviral PLA_2_ domains (25).

New capsids can generally be divided into two categories: natural capsids isolated from various sources and engineered capsids developed through processes like directed evolution or rational design. While engineering approaches have yielded promising capsids, screening and validation of these capsids require significant wet lab resources and typically focus on extensive validation from a given natural viral chassis or repeated iteration off of one (e.g., site saturation mutagenesis or spread out low diversity of a single isolate). This provides ample training data for these vector backbones for engineering; however, the evolved capsids must evolve from within the local minima of the parental vector which may require many steps of iterative engineering and refinement. Given nature has been sampling vector sequence space since before the existence of humans, it is reasonable to infer that there may be many unidentified vector chassis that would serve as a beneficial starting point for vector engineering since those vectors must assemble, package, and efficiently infect their hosts to propagate for eons.

Advances in computing power have made it feasible to explore and model the AAV capsid sequence space *in silico* (45, 46). However, considerable resources are still required both for the generation of training data and for experimental validation, and these models are typically restricted to the chassis of the training data (e.g., being variable region or serotype specific) and may not generalize. Furthermore, certain aspects (e.g., immune escape, viral assembly) of capsid design (46–48) are more easily addressed by these approaches than other more complex characteristics (e.g., multi-species cell specificity) . For generalizability, a wide region of sequence space must be covered in the training data representing multiple global and local optima; natural sequences are a perfect starting point for such training data with regard to expanding the currently under-sampled global optima in future machine learning approaches. Thus, a hybrid approach fusing model-based capsid engineering and capsid mining is likely not far off.

While two-thirds of novel MADCAP AAVs bound human IVIG, this binding was not neutralizing and did not interfere with cell transduction. Characterizing differences in the B-cell epitopes between AAVs could provide insights into the differences between binding and neutralizing antibodies. Of particular interest, MC46 (an AAV5-like virion) is more like both AAV5 and AAV-Go.1 (93.4% and 97.2%, respectively) than MC55 is to AAV7 (83.7%); however, of the two MC55 remains neutralizable unlike MC46. Furthermore, the variants of MC55 on the capsid surface are far more extensive than for MC46 (especially notable in the fivefold region). From this naïve view, we’d expect that MC55 would, therefore, be more able to handle the challenge of human IVIG neutralization, but that is not what is observed. This highlights that identity to a known isolate is not the only indicator of escape from neutralizability. The reason for this can be twofold: first just because a virus remains unknown to us does not mean it is not widely disseminated in the human population and generating NAbs; and second, MC55 as a relative to AAV7 remains much closer to the widely circulating human variants and, therefore, is more likely to be exposed to broadly neutralizing cross reactive antibodies those isolates generate.

The structural difference between binding and neutralizing antibodies can arise from distinct causes which are not mutually exclusive. First, antibodies can block essential interactions for a virus to prevent infection (49) and such interaction may be essential for one virus but not the other. Second, variants of the viral surface may alter or ablate binding kinetics or IgG binding geometry altering the ability of such immunoglobulins to block essential viral functions (29, 50). Finally, binding and neutralizing antibodies may be separate within the same viral pool (51, 52), in which case escape of a small part of the IVIG pool would lead to neutralization but not binding escape. *In silico* predictions suggest that the MADCAP AAVs do not share the same T-cell epitopes of natural isolates. However, we can’t preclude the possibility that they could still elicit T-cell immunogenicity through non-canonical T-cell epitopes which have yet to be characterized.

By leveraging the vast quantity of available SRA data, we have uncovered 139 new AAV capsids, greatly expanding the repertoire of known AAVs. We have identified capsids that resist neutralization by pre-existing human antibodies while maintaining the ability to transduce multiple regions of the primate brain following systemic delivery. These capsids have the potential to address pre-existing immunity, one of the most significant challenges in AAV gene therapy and pave the way for more effective and accessible gene therapies.

## MATERIALS AND METHODS

### Mining for novel AAVs and *Parvoviridae* capsids using MADCAP pipeline

#### Pre-screening

SRAs were pre-filtered to remove those that did not contain any *Parvoviridae* sequences. For SRAs published in or before December 2020, SRAs with parvoviridae scores greater than 10 were selected from Serratus database results (17). For SRAs published after December 2020, SRAs fastqs were mapped to a pan-*Parvoviridae* reference genome using bwa and only SRAs with detectable *Parvoviridae* reads were selected for downstream analysis.

#### Contig assembly and selection

For SRAs in which *Parvoviridae* reads were detected, *de novo* contig assembly was performed using Cenote-Taker 2 and contigs containing viral hallmark genes or features were identified. Contigs containing *Parvoviridae* hallmark gene sequences (non-structural or capsid proteins) were then further down selected based on length (>1000 nucleotides).

#### Identification of novel AAV capsids (MADCAP) from contigs

Putative full-length (length > 683 amino acids) AAV-like capsid sequences are selected, and novel sequences are identified using blast analyses. Briefly, we used BLAST to compare the mined sequences against the pataa and nr databases on NCBI BLASTp with a max target sequences of 10. We then take any sequence with at least one edit distance away from the sequence from NCBI BLASTp with the highest %identity in the aligned region as a MADCAP sequence. Phylogenetic analysis is then used to compare these sequences to a curated list of 162 known natural isolates (Table S4) (4). The closest known natural isolate is identified based on sequence identity (Table S5).

### Structural analysis of novel AAV capsids

Putative capsid VP1 sequences were folded by the open source Boltz-2 algorithm (53). Models were folded 25 times independently using MSA; for an estimate of robustness, the mean and individual measures were plotted, and at crossover point of pLDDT = 40 was used to define the PLA_2_ and VP3 core domains for analysis. The best-ranked model was used for figure generation and subsequent analysis. Structure figure images were made with ChimeraX (54). The structures for AAV5 and AAV7 used as references were 7KP3 and 7L5U,(55) respectively (56).

### Negative stain transmission electron microscopy

Purified vector was sent to Packgene for negative stain TEM service to confirm virion assembly and packaging of full virions. Briefly, 10 µL of AAV was placed on a carbon-coated Formvar support film, blotted dry, and stained with 2% uranyl acetate. Excess stain was removed with filter paper and imaged on an electron microscope on-site.

### Synthesis of ITR-capsid plasmids

AAV2 ITR plasmids with the mined or natural isolate capsid sequences were synthesized by Twist Bioscience. Single-stranded ITR acceptor plasmid encoding a CMV promoter, restriction enzyme sites, and BGH poly A sequence was digested with BstBI and PstI. Capsid sequences were synthesized to include the mined capsid sequence, AAV2 3′ UTR, barcode amplification section, and individual capsid barcode. The linearized ITR plasmid backbone was assembled via Gibson Assembly with synthesized capsid sequence insert with Twist Bioscience proprietary Stbl3-like cells. Sequence-verified ITR-capsid plasmids containing a unique mined capsid sequence and barcode were used for AAV manufacturing.

### Synthesis of Rep 3 STOP plasmid

AAVHBKO (57) RepCap plasmid was digested with SwaI and PaeI, and gene fragment encoding a truncated AAVHBKO capsid (516 bp deletion in VP3) sequence with stop codons after the VP1, VP2, and VP3 start sites was inserted via Gibson assembly using NEBuilder HIFi DNA assembly (New England Biolabs, E2621L). A sequence-verified clone was used for AAV manufacturing.

### Manufacturing of AAV pool

ITR-capsid plasmids were reconstituted in TE buffer to be 40 ng/µL and pooled at equi-volumetric ratios to create an ITR-capsid plasmid pool. AAVs were manufactured with a quadruple transfection method. VPC2.0 cells (Gibco, A35347) maintained in suspension culture at 4-6E6 cells per mL at 37°C, 8% carbon dioxide, 90% humidity at 110 RPM were diluted to 3.33E6 cells/mL for transfection in 500 mL (*n* = 10). VPC2.0 cells were transfected using the following the AAV-MAX Transfection kit (Gibco, A50515) protocol with Adhelper (Aldevron) (167.1 µg), Rep 3 STOP plasmid (488.2 µg), pUC19 (Thermo Fisher, SD0061) (31.8 µg), and ITR-capsid plasmid pool (5.16 µg corresponding to 500 copies/cell).

Viruses were purified from the media and cell pellet 68–72 h after transfection. Cells were separated from the media via centrifugation at 1,200 × *g* for 15 min and resuspended in 6 mL of AAV lysis buffer followed by three cycles of freeze-thaw. Cell lysate was separated from cell debris by centrifugation at 4,000 × *g* for 30 min. One-hundred milliliters of 40% Poly Ethylene Glycol 8000 (PEG 8000 Sigma Aldrich, 89510-1KG-F) was added to 500 mL of media incubated for 2 h on ice and centrifuged at 4,000 × *g* for 30 min. PEG precipitate was resuspended in 6 mL AAV lysis buffer. Cell lysate and PEG resuspension were combined and AAVs were purified with an iodixanol gradient (14 mL lysate, 9 mL 15% iodixanol, 6 mL of 25% iodixanol, 5 mL of 40% iodixanol, and 5 mL of 60% iodixanol). The purified virus was separated from the solution via centrifugation at 504,000 × *g* for 70 min and resuspended in 1× PBS (Gibco, 10010023).

For AAV pools that were used for *in vivo* studies, the iodixanol-purified AAVs went through further Cesium Chloride (CsCl) purification. CsCl (Sigma, 74687) was added to the post-iodixanol purified sample at 1.375 g/mL. The full virus population was separated from the solution via centrifugation at 366,500 × *g* for 25 h. Final purified virus suspended in 1× PBS with 0.005% pluronic acid F-68 (Thermo Fisher, 24040032) was concentrated with Amicon Ultra-4 centrifugal filters-100K (Millipore Sigma UFC810024).

### Manufacturing of rAAV

Recombinant AAVs (rAAVs) were manufactured using the triple transfection method with MADCAP or natural isolate capsid sequence RepCap plasmids and an ITR-CMV-LacZ-SV40-ITR transgene plasmid. Briefly, at an 85 mL scale, VPC2.0 cells were transfected following the AAV-MAX Transfection kit protocol with Adhelper (Aldevron) (24.2 µg), RepCap plasmid (72.9 µg), and transgene (30.4 µg). Viruses were purified from the media and cell pellet 68–72 hours after transfection, with PEG precipitation and an iodixonol gradient as in the viral pool. Final purified virus was separated in 1× PBS with 0.005% pluronic acid F-68 and concentrated as in the viral pool.

### Titration of AAV vectors

Titration of AAV pool was done by a Taqman (Thermo Fisher, 4304437) quantitative RT-PCR based assay using the following primer and probe sequences: T-BGH-F, 5′-TCTAGTTGCCAGCCATCTGTTGT-3′; T-BGH-R, 5′-TGGGAGTGGCACCCCA-3′; T-BGH-PB, 5'-/56-FAM/TCCCCCGTGCCTTCCTTGACC/36-TAMNph/3'.

### AAV library pooling for *in vivo* studies

AAV pools of mined capsids and natural capsids AAV1-10 were manufactured, purified, and titered separately. AAV pools of mined capsids and natural capsids were mixed at a 9:1 ratio of total vg for mouse IV injection. AAV pools of mine capsids and natural capsids were mixed at a 96.5:3.5 ratio of total vg for NHP IV injection. The viral concentration was brought to the specific injection concentration by dilution with 1× PBS 0.005% pluronic acid F-68.

### Animal and human samples

All experimental procedures involving animals were performed in full compliance with applicable institutional, national, and international regulations governing the humane care and use of laboratory animals. Serum samples from healthy human donors were obtained as cataloged items from Stem Cell Technologies using IRB-approved consent forms and protocols. NHP studies were performed at Labcorp. Only purpose-bred naïve cynomolgus macaques (male and female, 2–3 years old, 2–3 kg) were used in studies. NHPs were prescreened for AAV neutralizing antibodies, and seronegative animals were selected for the studies.

### *In vivo* experiment: mouse intravenous injection whole body transduction

Six female C57BL/6J mice were dosed by intravenous injection with 2E12 vg in 200 µL with the AAV pool.

Animals were euthanized on day 28. The brain, spinal cord, triceps, quadriceps, diaphragm, heart, pancreas, lungs, eye, gastrocnemius, kidneys, lymph nodes, soleus, spleen, tongue, skin, and liver were isolated into three sections and flash-frozen at −80°C.

### *In vivo* experiment: NHP intravenous injection whole body transduction

#### Intravenous injection in NHPs

Two cynomolgus macaques (Macaca fascicularis) were intravenously injected with an AAV pool of 5.54E13 vg/kg at an approximate dose volume of 4 mL/kg. All animals received a dose of IdeS of 25,000 units/kg at a concentration of 25,000 units/mL in PBS on day 0. All animals were injected with the AAV pool on day 3 via intravenous injection.

#### Intravenous injection

A butterfly catheter was primed by aspirating 1.5 mL in 1× PBS with 0.005% pluronic acid F-68 into a syringe connected to the catheter. This syringe used to prime the line remained connected to the catheter until the syringe with the AAV pool was primed. The syringe with the AAV pool was primed with the entire AAV pool volume. The AAV pool was aspirated and dispensed back into the storage vial at least two times with an 18-gauge needle attached to the dosing syringe. After the last priming step, the syringe was filled with the appropriate amount for dosing. For dosing, the syringe with 1× PBS with 0.005% pluronic acid F-68 was disconnected from the catheter to allow the line to drain, the needle was removed from the AAV pool syringe, and the primed AAV pool syringe was connected to the primed catheter. The AAV pool was dosed at over approximately 3 min via manual injection. The AAV pool dose was followed by a 1.5 mL flush of saline for injection administered directly into the catheter to flush the remaining AAV pool into the animal.

#### Blood collection

All animals were subjected to a blood sample collection (1.5 mL target) from the femoral vein on day 1 prior to dosing and day 29 prior to sacrifice. Blood samples were held at room temperature and allowed to clot prior to centrifugation. Samples were centrifuged within 1 h of collection, and serum was harvested into two approximately equal aliquots. Following harvesting, samples were placed on dry ice until stored at −80°C.

All animals were euthanized on day 29. Five 3-mm biopsy punches (Integra Lifesciences 33-32-P/25) were collected from each location: adrenal (left and right), aorta, cecum, colon, diaphragm, dose site, duodenum, epididymis (left and right), esophagus, gall bladder (drained), heart (atria and ventricles [left and right], and interventricular septum), ileum, jejunum, kidney (cortex and medulla [left and right]), liver (lateral lobe [left] and median lobe [right]), lungs (apical [left] and caudal [right]), lymph node (deep cervical and mandibular [left and right], and mesenteric), muscles (biceps femoris [right], intercostal [right], tibialis [right], triceps [right], quadriceps [right]), nerves (radial distal [right], radial proximal [right], sciatic distal [right], sciatic proximal [right]), optic nerve (left and right), pancreas, prostate, rectum, salivary gland mandibular (left and right), seminal vesicles, skin (hind limb and periorbital), spinal cord (cervical, lumbar, sacral, thoracic, cranial), stomach (body), testis (left and right), thymus, thyroid (left and right), tongue, trachea, and urinary bladder.

The brain was cut in a brain matrix at 4 mm coronal slice thickness with up to two 3-mm biopsy punches collected from each structure both the left and right hemispheres: frontal cortex, cingulate cortex, insular cortex, temporal cortex, caudate, putamen, globus pallidus, motor cortex, sensory cortex, thalamus, subthalamic nucleus, substantia nigra, hippocampus, entorhinal cortex, midbrain/pons, parietal cortex, cerebellum, cerebellar dentate, medulla, occipital cortex, corpus callosum, internal capsule, anterior commissure, centrum semiovale, cerebral peduncle, brachium pontis, medulla pyramids, and cerebellum arbor vitae.

The dorsal root ganglion was collected whole with up to five ganglia from the left and right sides: cervical, lumbar, sacral, thoracic, and trigeminal.

The brain, dorsal root ganglia, trigeminal ganglia, and liver samples were collected into separate nuclease-free/RNase-free/low binding tissue disruptor tubes with 1.4 mm beads (Fisherbrand, 15555799). Tissue samples were collected into separate nuclease-free/RNase-free/low binding tissue disruptor tubes with 2.8 mm beads (Fisherbrand, 15565799). All tissue samples were flash-frozen at −80°C.

### DNA/RNA isolation and cDNA synthesis

Animal tissues were homogenized, and nucleic acids were extracted as indicated (Table S6). RNA quality was measured with the TapeStation RNA kit (Agilent, 50675576, 50675577, 50675578) on a TapeStation 4200 (Agilent, G2991AA). RNA was concentrated with the RNeasy MinElute Cleanup kit (Qiagen, 74204). RNA concentration was measured with the RNA BR assay kit (Thermo Fisher, Q10210) on a Qubit. cDNA was synthesized from the concentrated RNA using the SuperScript IV First-Strand Synthesis System (Thermo Fisher, 18091050) with DNase (Thermo Fisher, 11766051) and the BGH specific primer OL_885: 5′- TGCAATTTCCTCATTTTATT-3′.

### Next-generation sequencing of unique barcodes

Unique barcodes corresponding to specific capsids were sequenced from plasmid pools, AAV pools, DNA, and cDNA from animal tissues.

Barcodes from plasmid or viral pools were amplified with the flanking primer OL_777 and OL_778 or OL_779 and OL_780 (Table S7).

Barcodes from 10 ng of the plasmid pools or exposed AAV transgenes from 5E9 vg of the AAV pool were amplified following the Phusion flash High Fidelity mastermix protocol (Thermo Fisher, F-548L). Five microliters of the PCR was barcoded with the NEBNext Multiplex Oligos for Illumina (New England BioLabs, E7600S) and Phusion flash master mix. Barcoded samples were pooled and purified with AMPure XP beads (Beckman Coulter, A63881). The sample pool was quality controlled with the TapeStation High Sensitivity DNA kit (Agilent, 50675587, 51906504, 50675584) on a TapeStation 4200 (Agilent, G2991AA) and concentration was measured with the Qubit dsDNA assay kit (Thermo Fisher, Q32851), diluted to 5 pM, denatured with 0.2 N NaOH, spiked with denatured 10% 5 pM PhiX (Illumina, 15017666) to be sequenced with a MiSeq v2 300 cycle reagent kit (Illumina, MS1022002).

Barcodes from DNA or RNA expressed transgenes in animal tissues were amplified with flanking primer sets OL_1094 with OL_1095 (Table S7).

Barcodes from 10 ng DNA or 5 µL cDNA of tissues were amplified following the Q5 High Fidelity Master Mix (New England Biolabs, M0492S). The PCR was purified using a 0.8× ratio of SPRIselect beads (Beckman Coulter, B23317). Up to 20 ng of the PCR purified product was barcoded with the NEBNext Multiplex Oligos for Illumina (New England BioLabs, E7600, E6440, or E6442) or and Q5 Mastermix. Barcoded samples were pooled and purified using a 0.7× ratio of SPRIselect beads. The sample pool was quality controlled for the presence of primers observed with the Tapestation High Sensitivity DNA kit (Agilent, 50675587, 51906504, 50675584) on a Tapestation 4200 (Agilent, G2991AA), and concentration was measured with the Qubit dsDNA assay kit (Thermo Fisher, Q32851). The sample pool was diluted to 550 pM and spiked with either 20% or 30% PhiX (Illumina, 15017666) to be sequenced with a NextSeq P1 or P2 300 cycle reagent kit (Illumina, 20050264, 20100985).

### NGS data analysis

To quantify barcodes in NGS readouts, FastQC was used to check the quality of each sample’s reads. A custom Python script was used to quantify the capsid specific barcodes from paired end. The normalized barcode count was calculated by the raw read counts of each barcode divided by the total read counts for each sample (plasmid, viral, tissue). This calculation was applied to plasmid (*F*_*p*_ = normalized plasmid counts in the library), viral (*F*_*v*_ = normalized viral counts in the library), and tissue (*F*_*t*_ = normalized counts in the tissue of interest) samples.

Manufacturability fitness was calculated for each AAV by $\frac{F_{v}}{F_{p}}$. Biodistribution and transduction fitness were calculated for each AAV for individual tissues by $\frac{F_{t}}{F_{v}}$. Fold change to AAV9 was calculated by $\frac{\frac{F_{t}}{F_{v}}}{{\left(\frac{F_{t}}{F_{v}}\right)}_{AAV9}}$. Graphs were made with both Python and GraphPad Prism 10.

NHP cerebral cortical region includes tissue samples from the motor, frontal, cingulate, sensory, occipital, insular, temporal, entorhinal, and parietal cortices. NHP subcortical region includes tissue samples from the hippocampus, thalamus, caudate, putamen, midbrain/pons, medulla pyramid, globus pallidus, subthalamic nucleus, substantia nigra, and medulla. NHP white matter region includes tissue samples from the corpus callosum, internal capsule, anterior commissure, centrum semiovale, cerebral peduncle, brachium pontis, and cerebellum arbor vitae. NHP spinal cord and DRG include samples from the cervical, thoracic, lumbar, and sacral regions. NHP skeletal muscle included samples from biceps femoris, tibialis, triceps, and quadriceps. NHP cardiac muscle includes samples from the atria, ventricles, and interventricular septum. Skin included samples from the hind limb and periorbital. Mouse skeletal muscles included tissue samples from the triceps and quadriceps.

### Cladogram generation

Cladograms were generated by utilizing iTOL as described in Letunic and Bork (58). Briefly, we used ClustalW to align 162 AAV natural isolates and 139 cap sequences from the MADCAP search and then used the Geneious tree builder in Geneious 10.2.6 (https://www.geneious.com) to make corresponding newick files (File S2 and S3). After uploading this newick file to iTOL, we used either circular mode or rectangular mode for plotting and ignored branch lengths to have an almost continuous circular representation for the tree.

### Brain heatmap representations

Brain heatmaps were generated utilizing the Macaque Brain Visualization Tool protocol described in Kondratova et al. (59). Briefly, normalized transduction fitness was normalized for DNA by the input of 10 ng starting material and was normalized for cDNA by the input ng amount into the barcoding PCR. Normalized biodistribution and transduction fitness were plotted with a five-color stepwise gradient (59).

### NAb assay and analysis

#### MOI screen

HEK293T cells were plated in a 96-well plate (5E4 cells/well) and were infected with Ad5/ts149 helper virus at an MOI of 1 and incubated at 39°C 5% CO_2_ for 4 h. Cells were then infected with individual MADCAP rAAVs or natural isolate rAAV controls (AAV2 and AAV9) at MOIs of 5E2, 5E3, 5E4, or 5E5 and incubated for 3 days at 39°C 5% CO_2_. MADCAP rAAV transduction and LacZ transgene production were quantified using Galacto-StarTM β-Galactosidase Reporter Gene Assay Kit (Thermo Fisher, T1014) in black 96-well plates (Greiner Bio-One, 655209). Luminescence was read on a BioTek Synergy 4 Microplate Reader (integration time 1 s, sensitivity 200).

#### NAb assay

HEK293T cells were infected with Ad5/ts149 helper virus at an MOI of 1 and incubated at 39°C 5% CO_2_ for 4 h. During helper virus incubation, MADCAP rAAVs and natural isolate controls were diluted to 2× MOI selected from the MOI screen (MOI of 5E3 or 5E4 for MADCAP rAAVs; MOI of 5E2 for AAV1; MOI of 1E2 for AAV2; MOI of 5E3 for AAV9). MADCAP rAAVs were incubated with an equal volume of serially diluted human IVIG (purified 5% gammaglobulin, Sigma, G4386) for 1 h at 37°C 5% CO_2_. After 4 h incubation with the helper virus, cells were infected with IVIG-incubated rAAVs and incubated for 3 days at 39°C 5% CO_2_. Transduction and LacZ transgene production were quantified using Galacto-StarTM β-Galactosidase Reporter Gene Assay Kit. Luminescence (RLU) was read on a BioTek Synergy 4 Microplate Reader (integration time 1 s, sensitivity 200).

Normalized RLU values were calculated by subtracting the average of the RLU from wells containing media only that were not treated with rAAV or IVIG. The percent neutralization for each AAV was calculated by % neutralization = 100 − {[(average normalized IVIG + rAAV RLU)/(average normalized rAAV only RLU)] × 100}.

For screening of individual healthy human donors, we obtained cataloged sera from de-identified, appropriately consented donors in accordance with IRB policies and procedures. Donors were screened for NAbs from major natural serotypes including AAV2 (MOI 1E2), AAV5 (MOI 1E3), AAV7 (MOI 1E3), and AAV9 (MOI 1E4). Seropositive donors were also screened for cross-reactive NAbs against MC55 (MOI 5E3) and MC46 (MOI 5E4), as described previously, with gene transfer detected by quantification of a LacZ transgene.

### Total antibody-binding assay

ELISA plates (Medisorp Plates, Thermo Fisher 467320) were coated with 5E9 VG rAAV diluted in 100 µL ELISA coating buffer (Bethyl Laboratories E107) per well and incubated overnight at 4°C. Plates were washed once (R&D Systems Wash Buffer 895126) using a BioTek ELISA washer, then blocked with 300 µL ELISA blocking buffer (1% Casein Blocker, Thermo 37528) + 0.05% Tween-20 per well for 1 h at 37°C. Purified human IVIG (Human Gammaglobulin, Sigma G4386) was serially diluted in blocking buffer for final dilutions of 1:10, 1:30, 1:90, 1:270, 1:810, 1:2,430, 1:7,290, and 1:21,870. After removing blocking buffer, serial dilutions of IVIG were added at a final volume of 100 µL per well and incubated for 2 h at 37°C. For IdeS controls, IVIG was pre-incubated with 1 unit of IdeS protease (Promega V7515) per 1 µg of IVIG. Plates were then washed four times with wash buffer. Secondary antibody (Goat Anti-Human IgG-Fc-HRP, Southern Biotech 2047-05) was diluted 1:5,000 in blocking buffer and 100 µL per well were incubated for 1 h at room temperature. Plates were then washed eight times in wash buffer. For colorimetric substrate conversion, 100 µL peroxidase substrate (KPL SureBlue Reserve TMB Microwell Peroxidase Substrate, SeraCare 5120-0080) was added per well and incubated for approximately 5 min until a color gradient was visible, followed by 100 µL stop solution (KPL TMB Stop Solution, SeraCare 5150-0021). Plates were read at 450 nm (Molecular Devices SpectraMax Plus). IVIG Kd values were calculated using nonlinear regression of OD450 binding curves.

### Mouse IVIG passive transfer

C57BL/6J mice were injected I.V. with single vector rAAVs (1E11 VG in 100 µL sterile H₂O per mouse) for MADCAP AAVs MC46 and MC55 and natural serotype control AAV9 (Table S8). For each vector group, five animals were injected I.V. with 9 mg (180 µL volume) pharmaceutical grade human IVIG 24 h prior to administration of AAV vectors. For IdeS controls, animals were injected retro-orbitally with 25 µg IdeS protease 30 min prior to administration of IVIG. Animals were euthanized on day 28 and liver, heart, brain, skeletal muscle (quadriceps and triceps), kidneys, spinal cord, and spleen were flash frozen at –80°C.

### Transgene biodistribution quantification

LacZ transgene was quantified from 500 ng of isolated DNA using a Taqman (Thermo Fisher, 4304437) qRT-PCR based assay using the following primer and probe sequences: SV40-F, 5′-AGCAATAGCATCACAAATTTCACAA-3′; SV40-R, 5′-GCAGACATGATAAGATACATTGATGAGTT-3′; SV40-PB, 5'-/56-FAM/AGCATTTTTTTCACTGCATTCTAGTTGTGGTTTGTC/36-TAMNph/3'.

### Mouse NAb analysis-gene transfer with pre-existing immunity

C57BL/6J mice were injected IM with single vector rAAV1-OVA or rAAVrh32.33-OVA (5E10 vg/mouse) and followed for at least 16 weeks, with serum collected weekly for NAb analysis. To quantify gene transfer of MC55 and MC46 in the presence of pre-existing neutralizing antibodies against AAV1 or AAVrh32.33, we performed neutralizing antibody assays in HEK293T cells using serum from rAAV1 or rAAVrh32.33 dosed-mice. To obtain adequate serum volume to assay multiple vectors, serum samples from weeks 10, 12, and 14 were pooled for each animal as these time points showed consistent levels of anti-capsid IgG by ELISA. Serially diluted serum from rAAV1-dosed mice was incubated with AAV1 (MOI 2E3), MC55 (MOI 5E3), or MC46 (MOI 5E4) vector and serially diluted serum from rAAVrh32.33-dosed mice was incubated with AAVrh32.33 (MOI 2.5E4), MC55 (MOI 5E3), or MC46 (MOI 5E4) vector as previously described and incubated with Ad5/ts149 infected HEK293 cells for 3 days. Gene transfer was quantified by detection of a LacZ transgene using Galacto-StarTM β-Galactosidase Reporter Gene Assay Kit.

## Data Availability

All data, code, and materials used in the analysis are available from the corresponding author upon reasonable request (gmu@sanofi.com).

## SUPPLEMENTAL MATERIAL

Supplemental figures jvi.00630-26-s0001.pdf Fig. S1 to S16.

Table S1 jvi.00630-26-s0002.csv Origin of MADCAP capsids.

Table S2 jvi.00630-26-s0003.csv Identity matrix of AAV2-like MADCAP capsids.

Supplemental tables jvi.00630-26-s0004.pdf Tables S3 and S6-S8.

Table S4 jvi.00630-26-s0005.xlsx Identity of 162 natural isolates used in the study.

Table S5 jvi.00630-26-s0006.csv Identity matrix of all capsids used in the study.

File S1 jvi.00630-26-s0007.pdf Full phylogenetic tree of capsids used in the study.

File S2 jvi.00630-26-s0008.newick Newick file for tree reconstruction.

File S3 jvi.00630-26-s0009.csv Newick file for tree reconstruction.

ASM does not own the copyrights to Supplemental Material that may be linked to, or accessed through, an article. The authors have granted ASM a non-exclusive, world-wide license to publish the Supplemental Material files. Please contact the corresponding author directly for reuse.
