# Supplementary material for: MADCAP: isolation of novel nAb-naïve AAV capsids from metagenomic data
Source: J Virol. 2026 Sep 9;100(10):e00630-26. doi: 10.1128/jvi.00630-26 (PMC13712814; doi:10.1128/jvi.00630-26)
Supplement: Supplementary file 1 [file jvi.00630-26-s0001.pdf]

Supplementary Materials for

MADCAP: Isolation of novel NAb-naïve AAV capsids from metagenomic data

Eugenia Lyashenko[1]†, Tess Torregrosa[1]†, Alexandra Ysasi[1]†, Jason Wu[1], Jie Bu[1], Margaret Hennessy[1], Youngji Na[1], Michael J Ryan[1], Mehmet Takar[1], Joshua A. Hull[1], Edith L Pfister[1], Christian Mueller[1], Sourav R. Choudhury[1]*
Corresponding author: gmu@sanofi.com

The PDF file includes:

Figs. S1 to S16

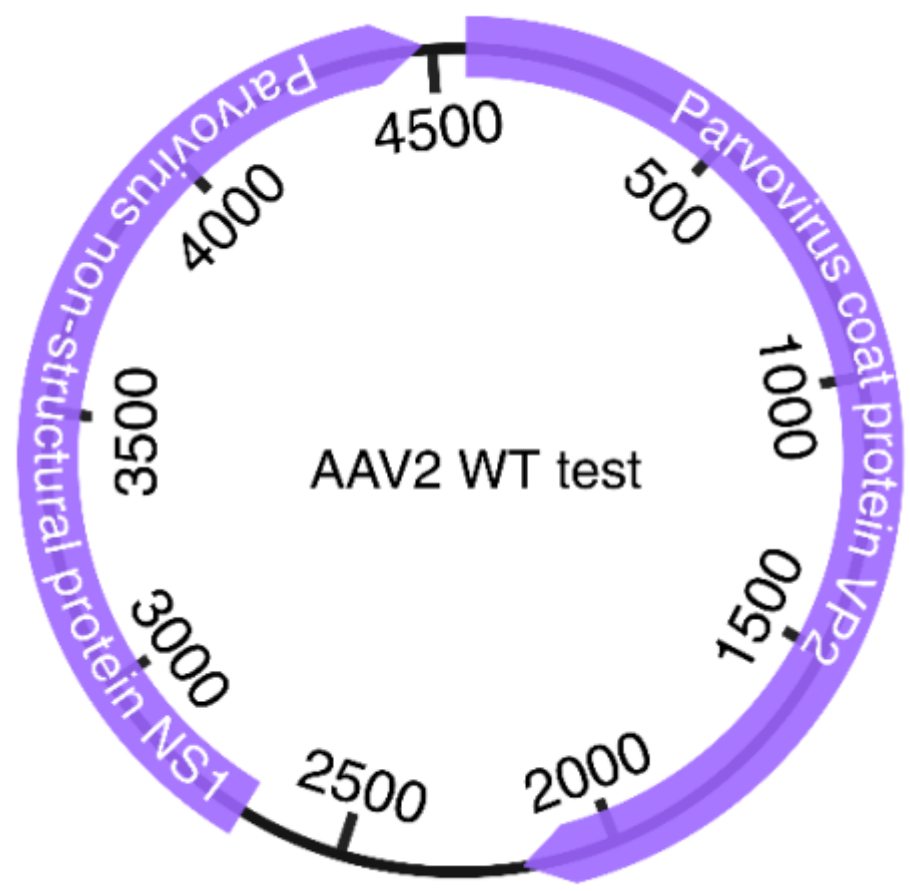

Figure S1. Validation of Cenote-Taker using a reference AAV2 genome.
Cenote-Taker identifies VP coat protein and non-structural (rep) protein of parvovirus in a wild-type AAV2 genome fasta sequence (NCBI Accession ID NC_001401). Cenote-Taker was run in annotation mode only. Command:"Cenotetaker3 -c NC_001401.fna -r test_ct4 -p F -am T".

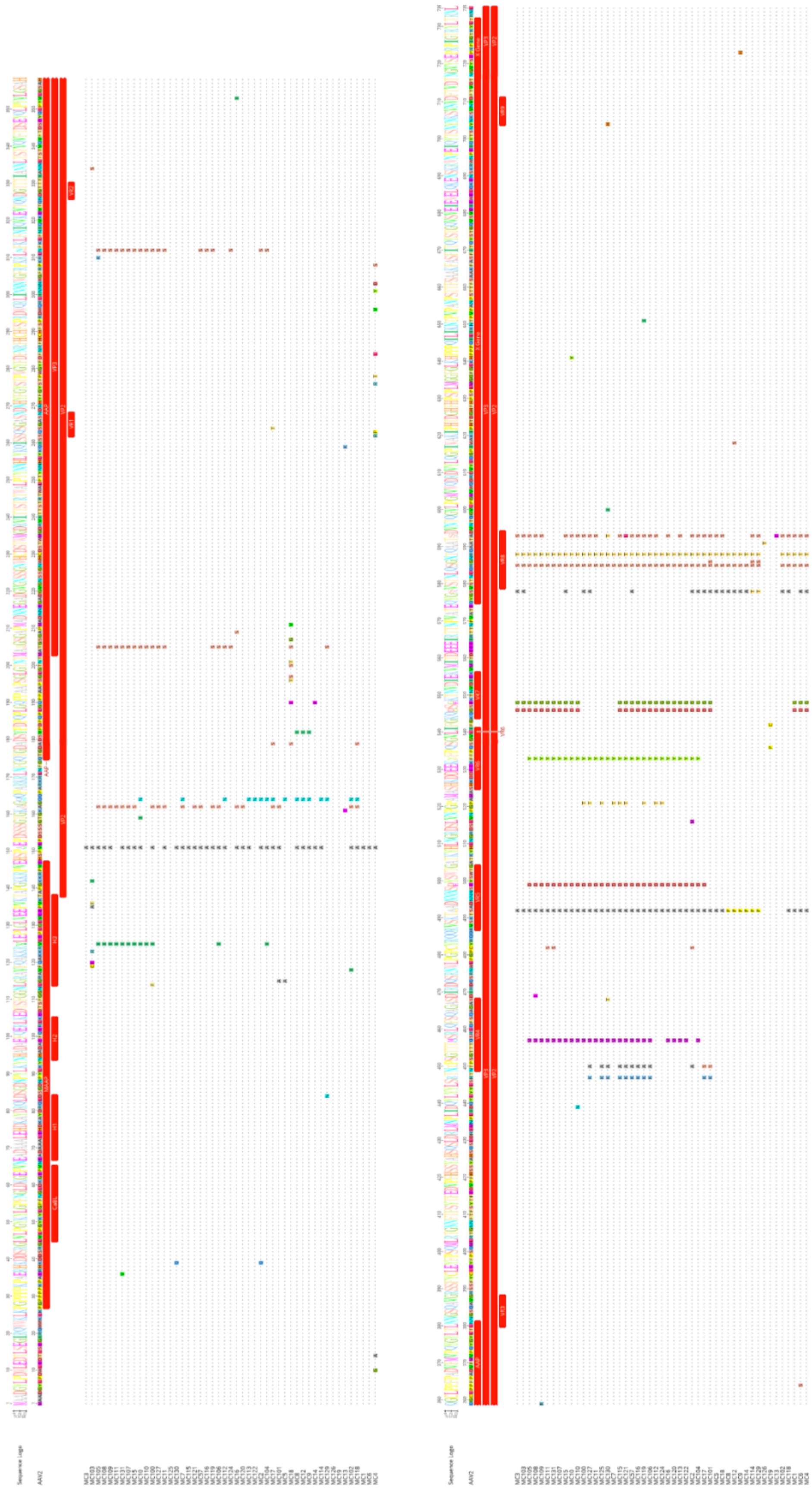

Figure S2. Amino acid changes in novel AAVs cluster in variable regions.

Multiple sequence alignment of AAV2-like MADCAP virions with AAV2.

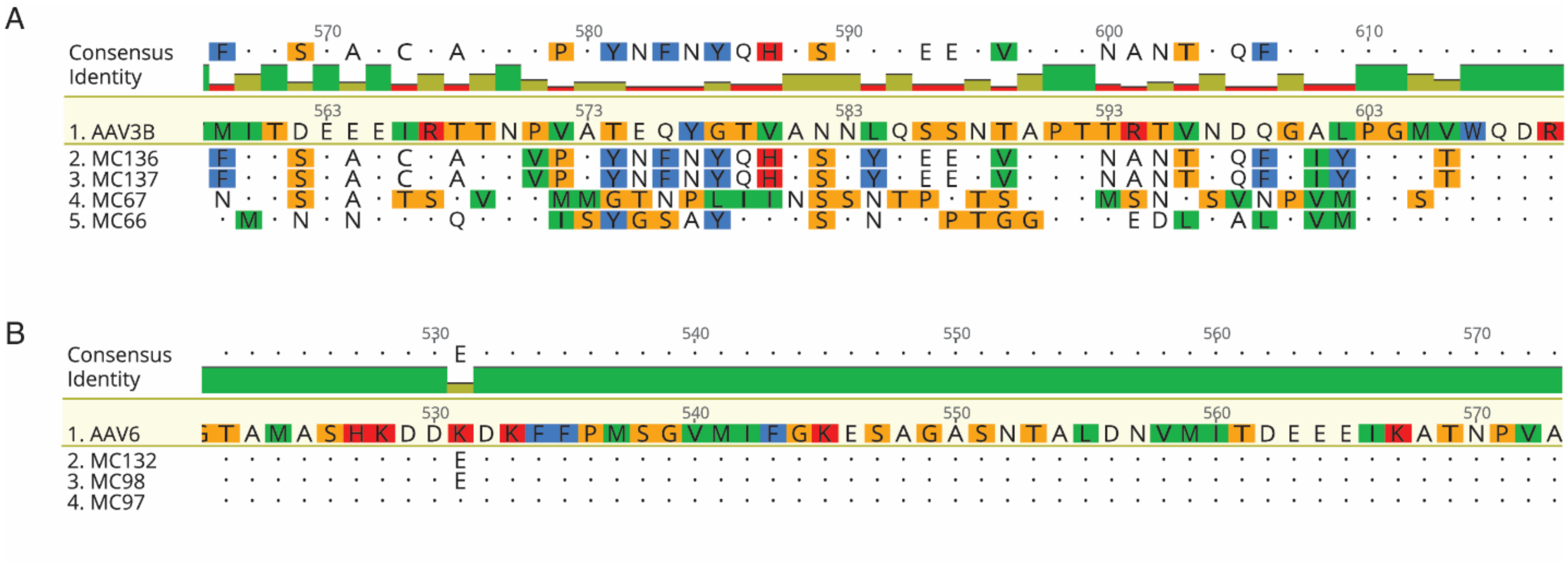

Figure S3. Sequence alignment of natural isolates and MADCAP AAV sequences.

(A) alignment of a region of AAV3B and AAV3B-like MADCAP virions proximal to AAV3B 594R responsible for HSPG binding in AAV3B. (B) alignment of a region of AAV6 and AAV6-like MADCAP virions proximal to AAV6 K531 responsible for HSPG binding in AAV6.

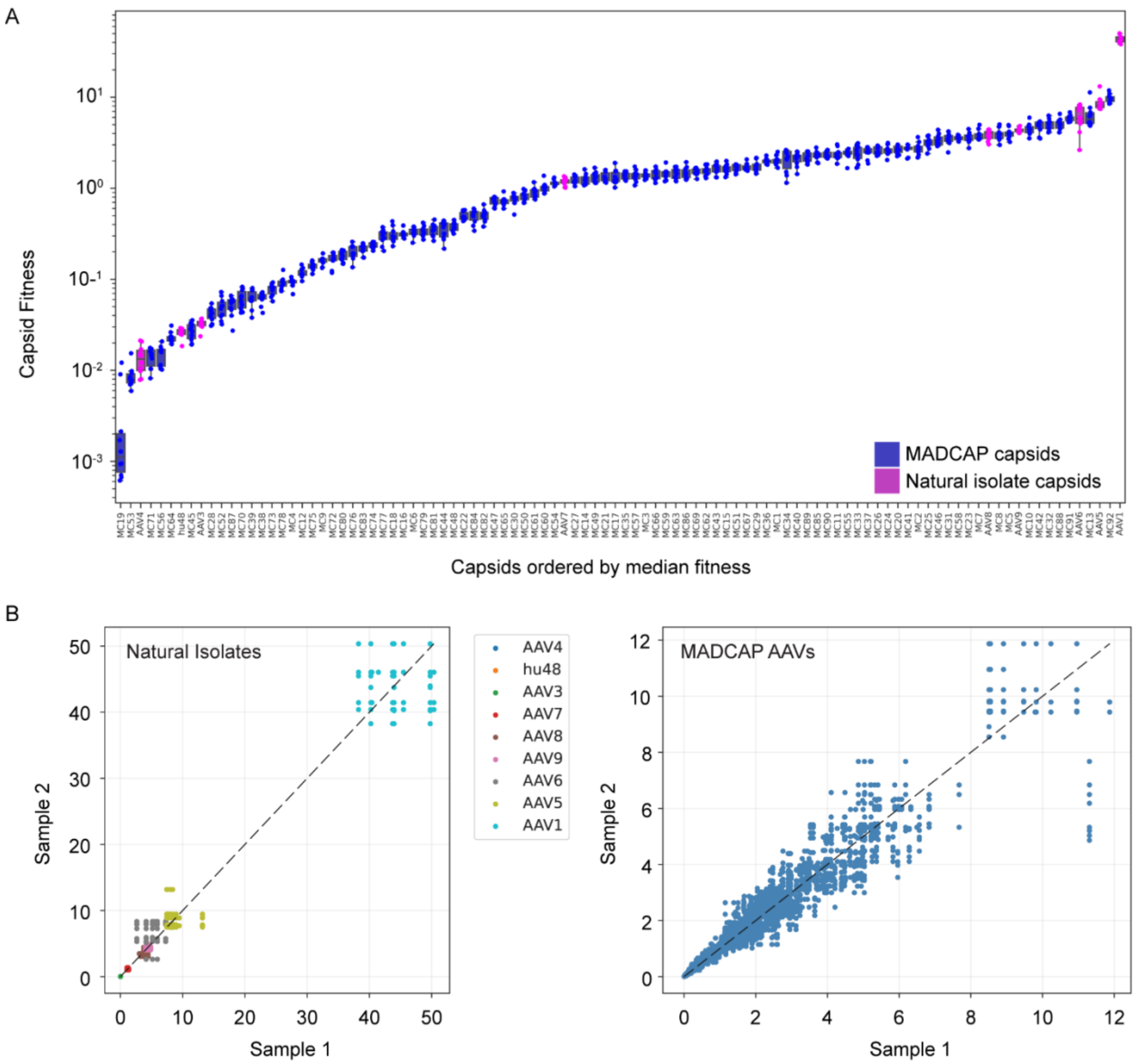

Figure S4. Novel AAVs successfully and reproducibly package.

(A) Quantification by next generation sequencing of AAV capsid pool. Individual ITR-capsid plasmids were pooled at equi-volumetric ratios and the pool was packaged. Novel capsids are shown in blue, while known natural isolates are shown in pink. (B) Correlation plots of independent packaging replicates for natural isolates and MADCAP AAVs.

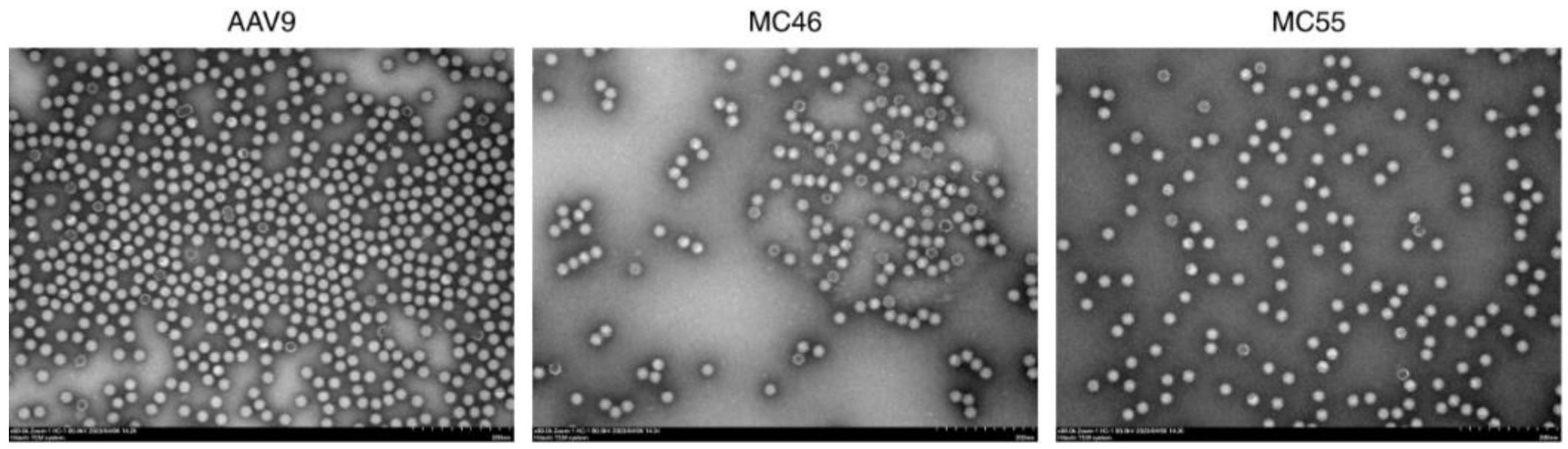

Figure S5. TEM images of AAV9, MC46, and MC55 viral capsids after AAV manufacturing and purification. For all virions, full particles are abundant and identifiable by their “filled in” appearance compared to empties which appear “hollow”; otherwise, particles demonstrate generally uniform spherical features expected of AAV.

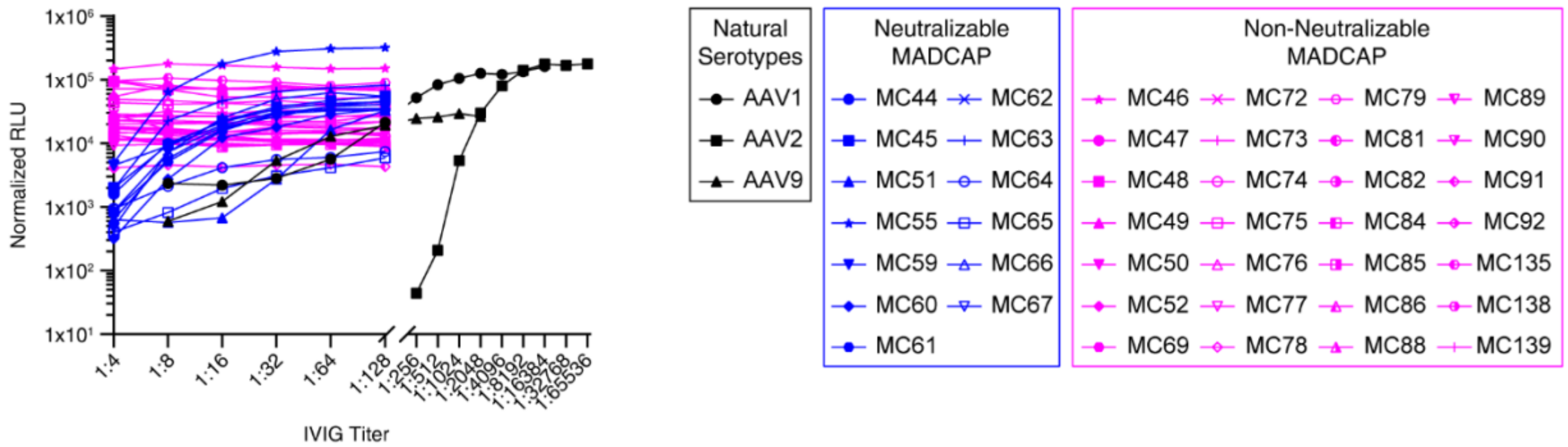

Figure S6. Many novel MADCAP AAVs lack pre-existing neutralizing antibodies in humans.

LacZ reporter expression in Ad5/ts149-infected HEK293 cells following transduction with individual recombinant vectors for 41 novel-clade MADCAP AAVs incubated with serial dilutions of human IVIG.

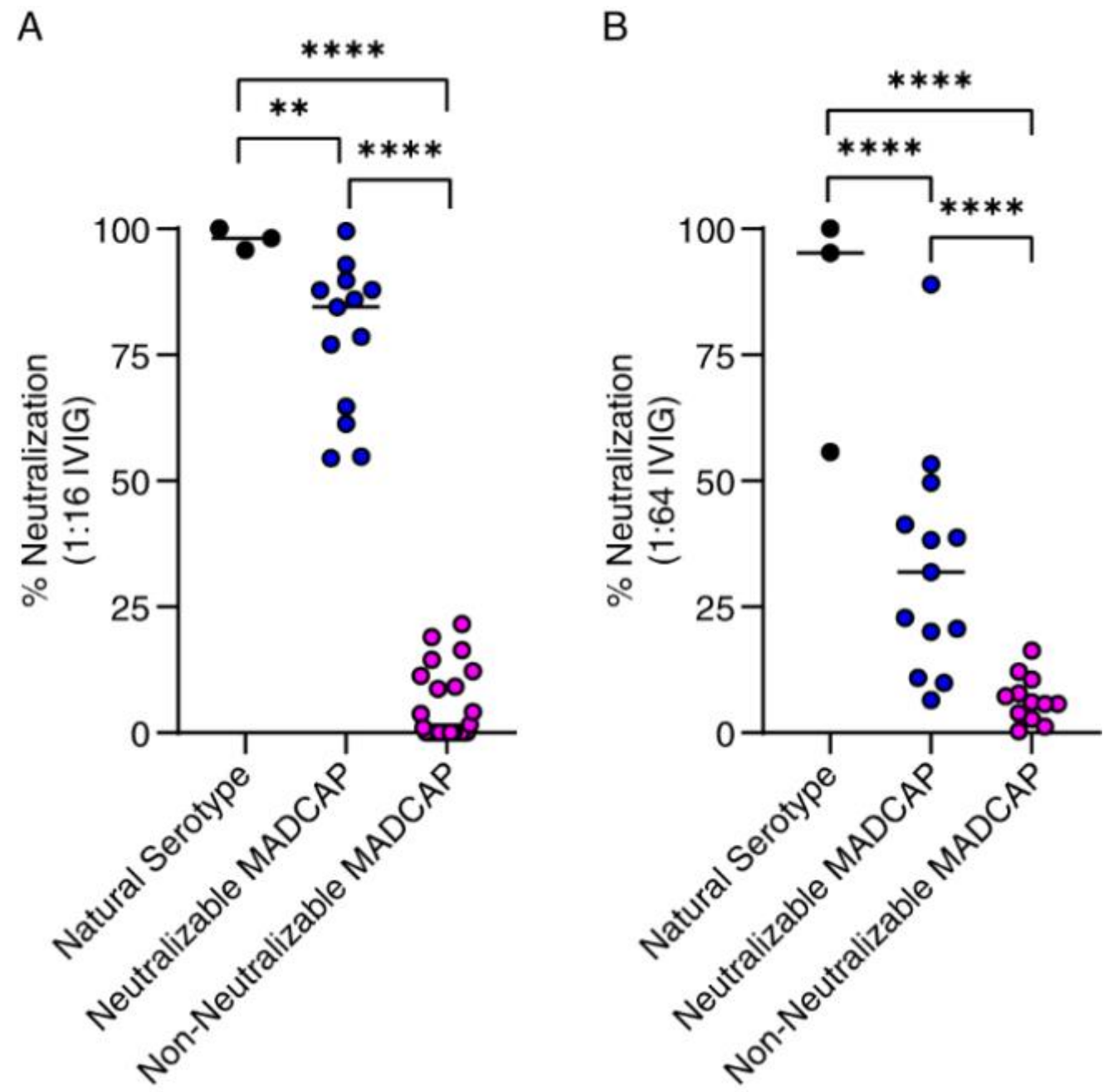

Figure S7. MADCAP AAVs show significant differences in neutralization across IVIG concentrations.

Percent neutralization at (A) 1:16 and (B) 1:64 dilutions of IVIG.

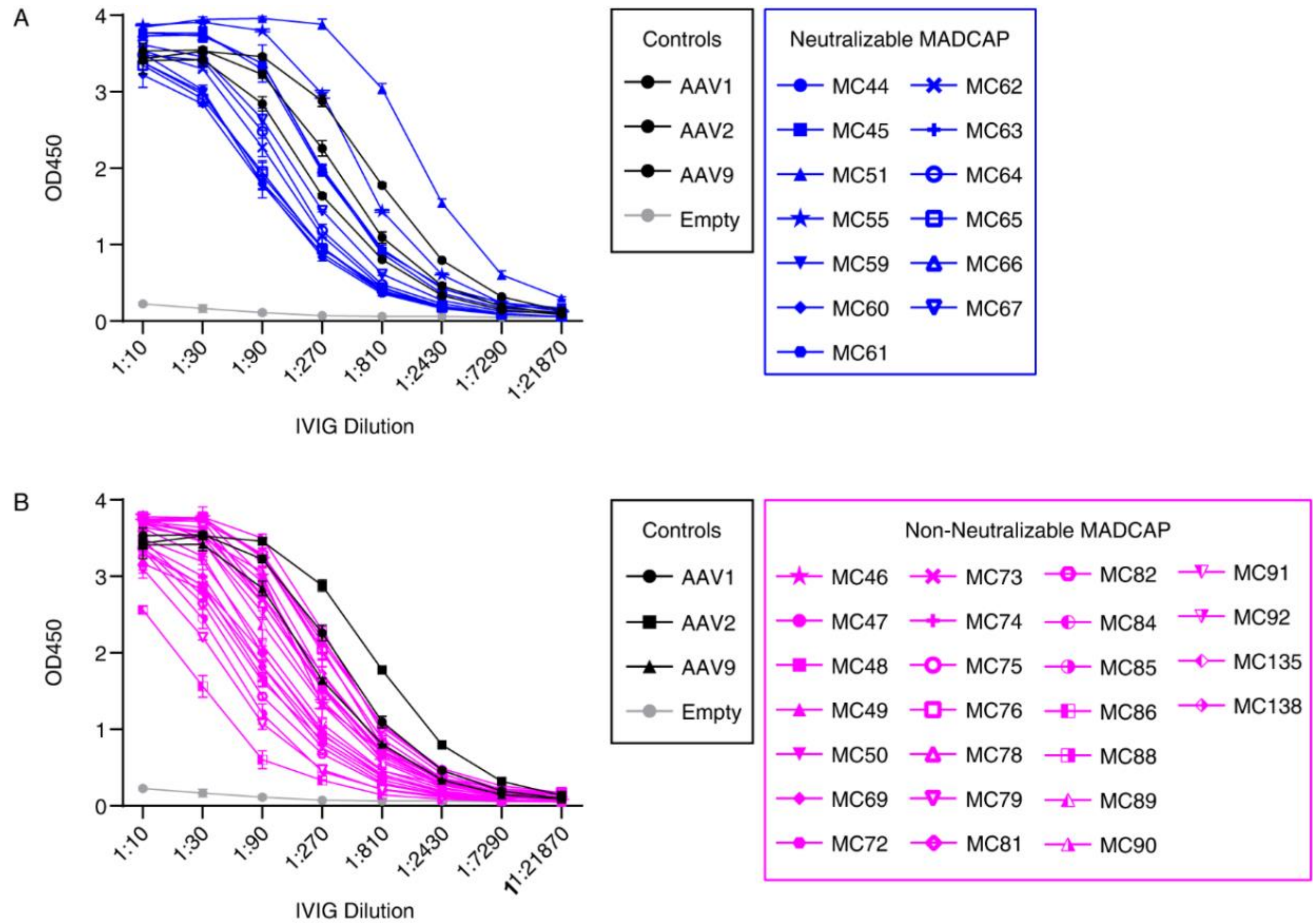

Figure S8. Total IVIG binding for natural serotype and MADCAP AAVs.

(A) Neutralizable (blue) and (B) non-neutralizable (magenta) MADCAP AAVs compared to natural serotype controls (black). Kd values shown in Figure 2D were calculated by nonlinear regression on OD450 binding curves.

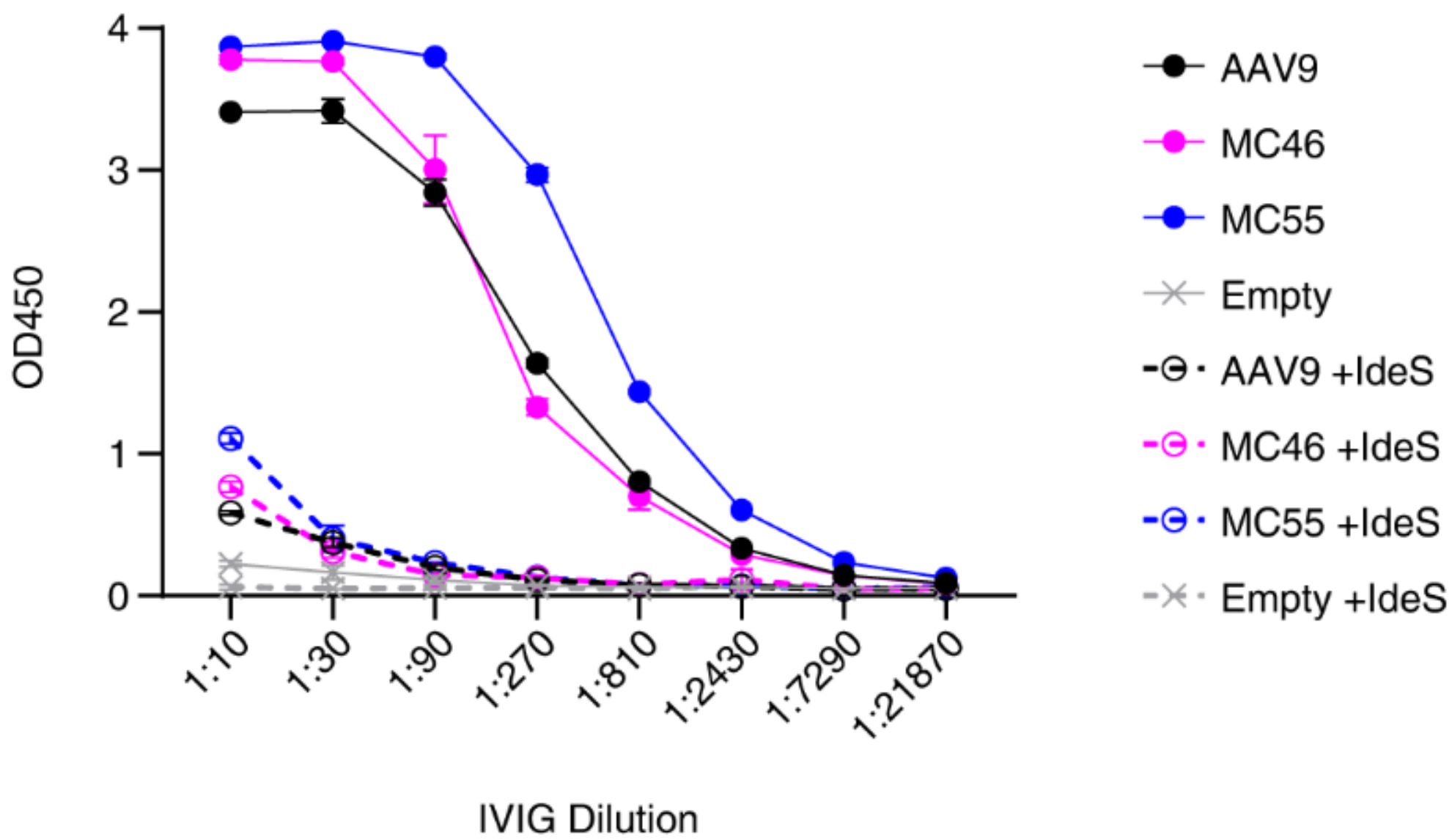

Figure S9. Total IVIG binding readout is IgG-specific.
Representative single vectors in the presence or absence of IdeS protease, which selectively cleaves human IgG, demonstrate differential antibody binding without IdeS.

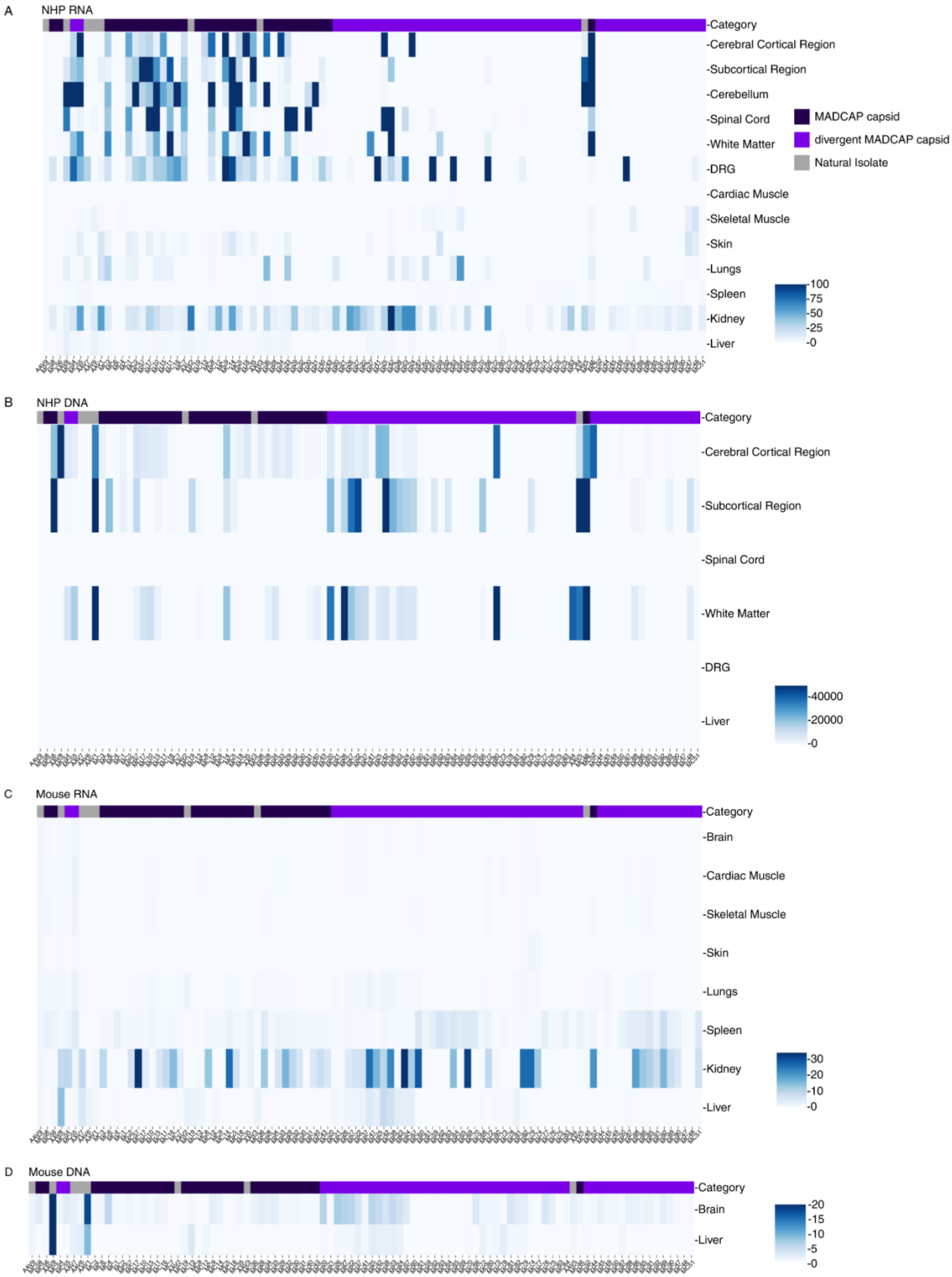
A
NHP RNA
-Category
-Cerebral Cortical Region
-Subcortical Region
-Cerebellum
-Spinal Cord
-White Matter
-DRG
-Cardiac Muscle
-Skeletal Muscle
-Skin
-Lungs
-Spleen
-Kidney
-Liver
MADCAP capsid
divergent MADCAP capsid
Natural Isolate
-100
-75
-50
-25
-0
B
NHP DNA
-Category
-Cerebral Cortical Region
-Subcortical Region
-Spinal Cord
-White Matter
-DRG
-Liver
-40000
-20000
-0
C
Mouse RNA
-Category
-Brain
-Cardiac Muscle
-Skeletal Muscle
-Skin
-Lungs
-Spleen
-Kidney
-Liver
-30
-20
-10
-0
D
Mouse DNA
-Category
-Brain
-Liver
-20
-15
-10
-5
-0

Figure S10. Several MADCAP capsids exhibit enhanced biodistribution and transduction profiles relative to AAV9 in CNS and peripheral tissues.

A pool of MADCAP capsids and natural serotypes AAV1-10 was intravenously injected into cynomolgus macaques (A,B) at a dose of 5.54E13 vg/kg or C57BL/6J mice at 2E12 vg/mouse (C,D) (A) Transduction profiles of MADCAP and natural isolates normalized to AAV9 transduction in CNS tissues from the cerebral cortical, subcortical, cerebellum, spinal cord, white matter, and DRG regions, and peripheral tissues from the skin, lungs, spleen, kidney, and liver regions. (B) Biodistribution profiles of MADCAP and natural isolates normalized to AAV9 biodistribution in CNS tissues from the cerebral cortical, subcortical, cerebellum, spinal cord, white matter, and DRG regions, and liver. (C) Transduction profiles of MADCAP and natural isolates normalized to AAV9 transduction in the brain, cardiac and skeletal muscles, skin, lungs, spleen, kidney, and liver. (D) Biodistribution profiles of MADCAP and natural isolates normalized to AAV9 biodistribution in the brain and liver.

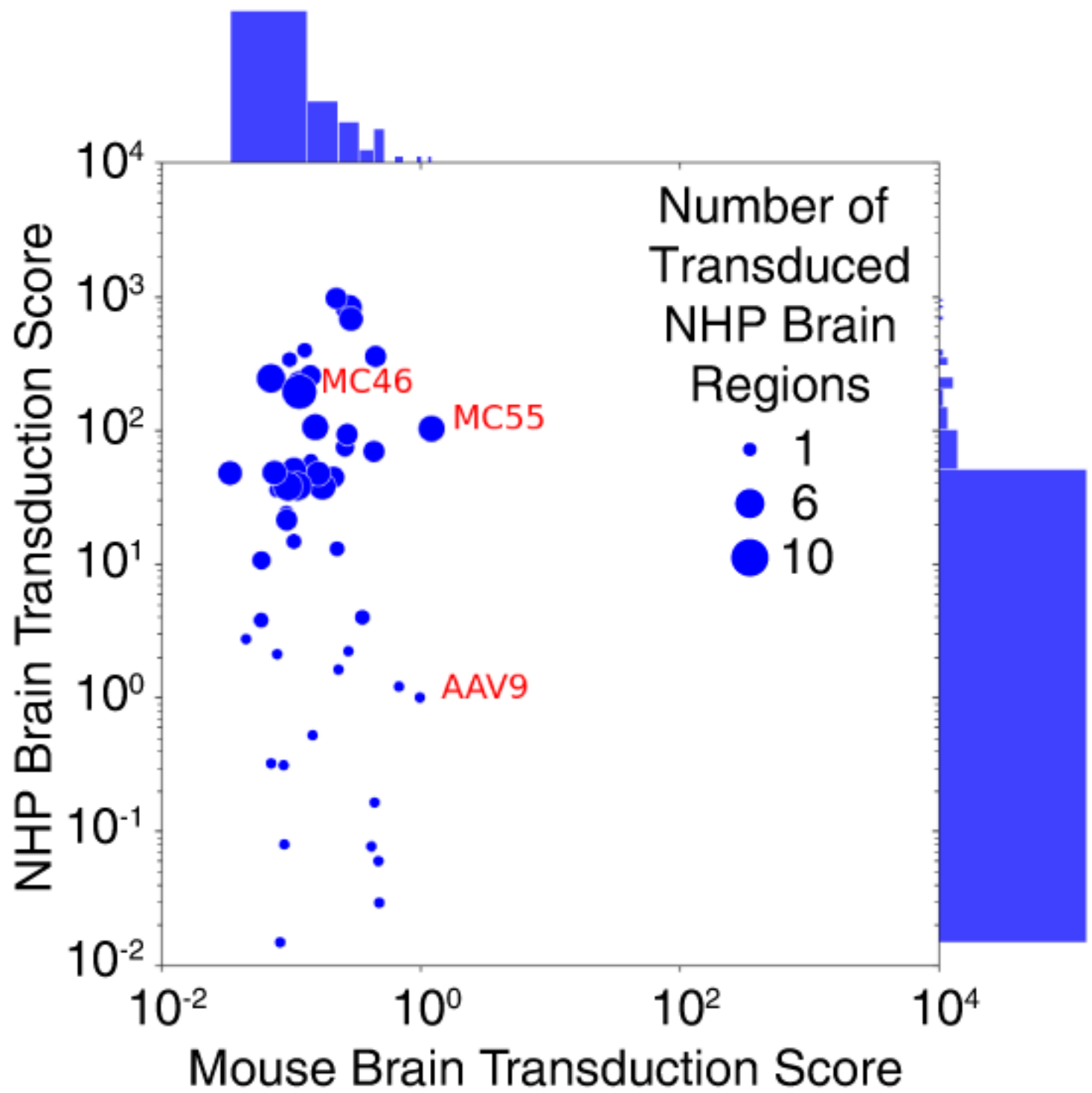

Figure S11. Correlation of mouse and NHP transduction normalized to AAV9.

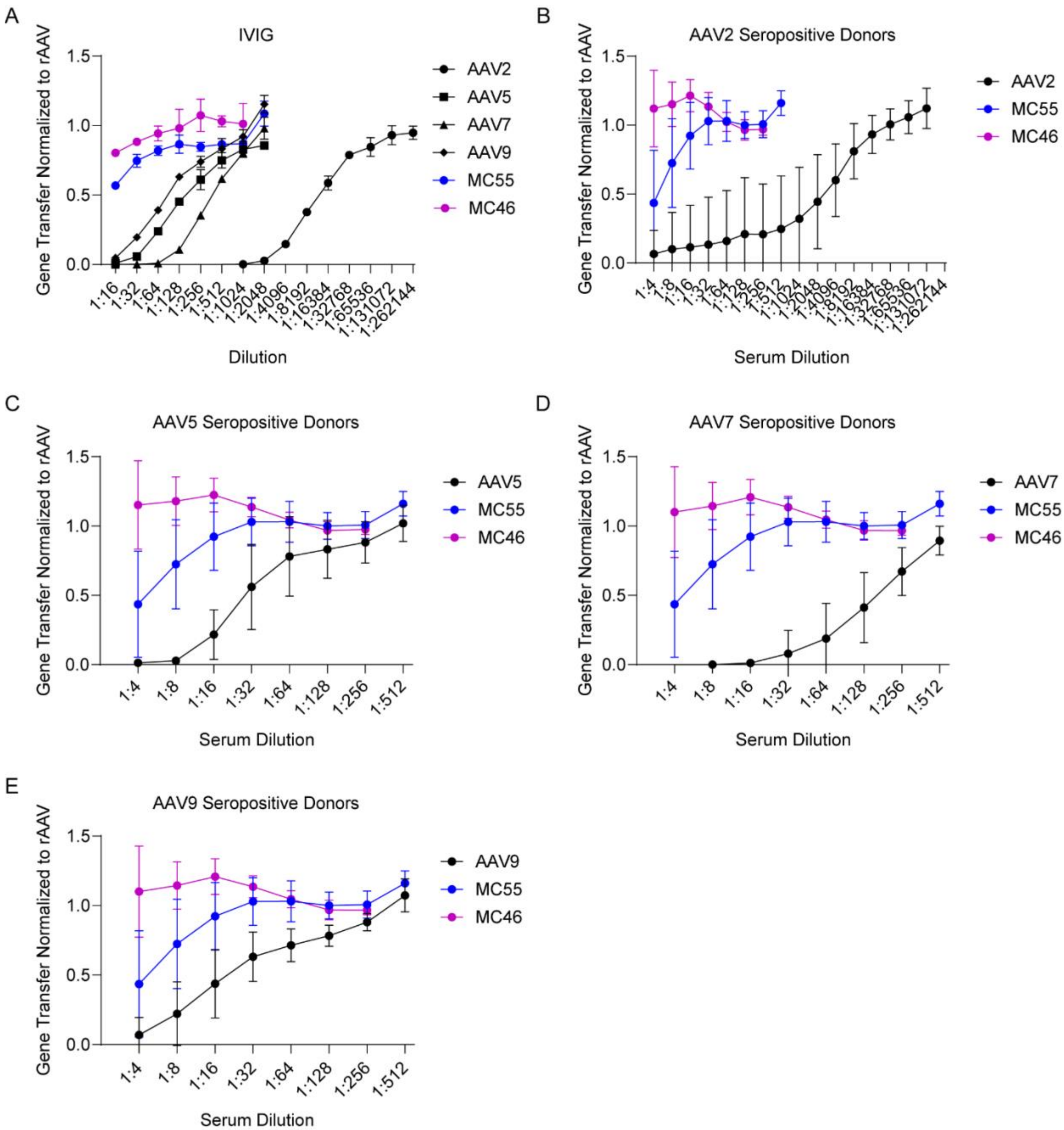

Figure S12. Gene transfer of select MADCAP vectors MC55 and MC46 relative to natural serotypes.

Gene transfer values at each IVIG/serum dilution are normalized to gene transfer with rAAV vector alone. (A) Population level comparison of MC55/MC46 versus natural serotypes using purified pool IVIG

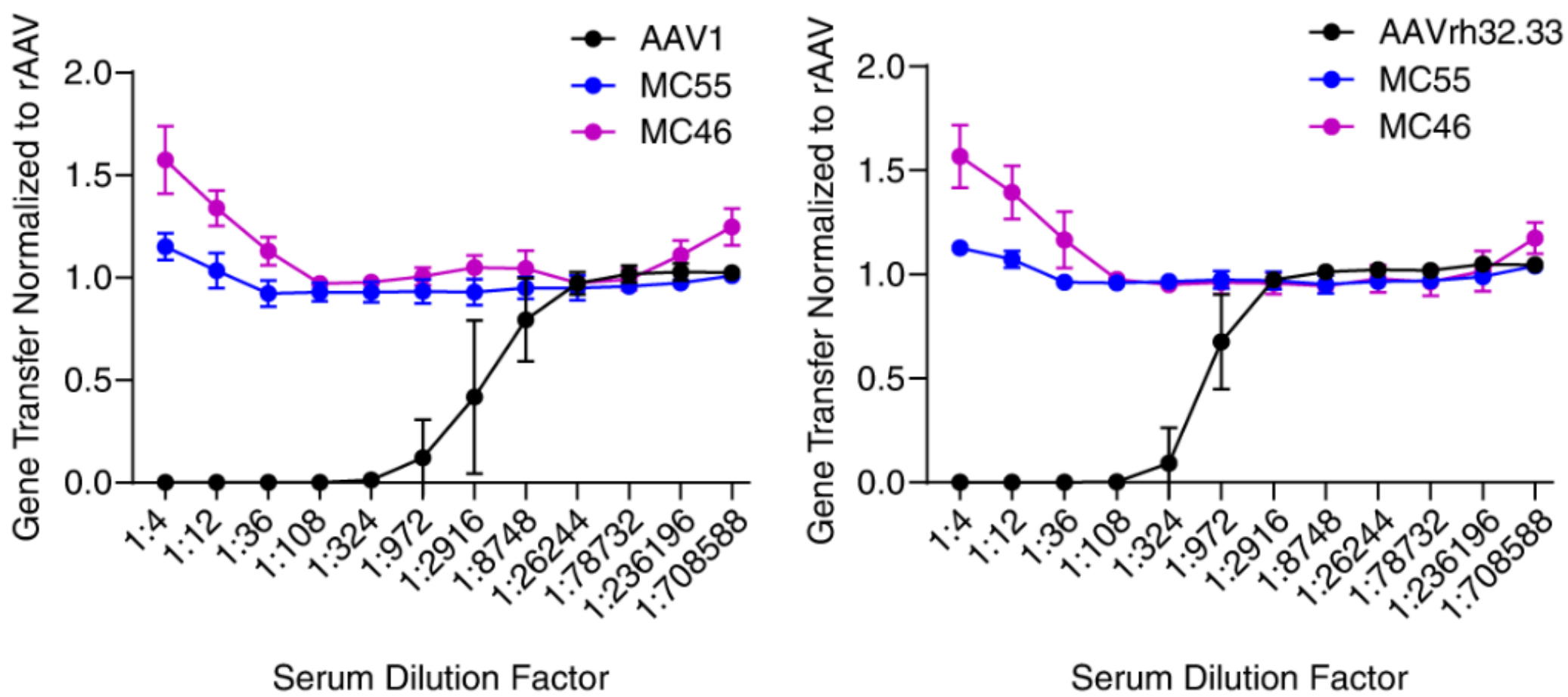

Figure S13. Gene transfer of MADCAP vectors MC55 and MC46 in mice previously treated with AAV1 or AAVrh32.33.

A

	SV40 DNA per 400ng total DNA				
	Replicate 1	Replicate 2	Replicate 3	Replicate 4	Replicate 5
AAV9	14962.372	6756.758	13053.887	14466.045	20559.314
AAV9+IVIG	1018.819	33.645	149.639	96.405	
AAV9+IVIG+Ides	4219.832	10563.367	4635.555	6326.648	
MC46	107.603	45.201	99.882	73.577	118.85
MC46+IVIG	761.349	292.063	202.399	92.656	88.329
MC55	3654.072	5587.464	4642.365	10309.318	
MC55+IVIG	1467.813	1667.719	1289.788	634.806	1352.24

B

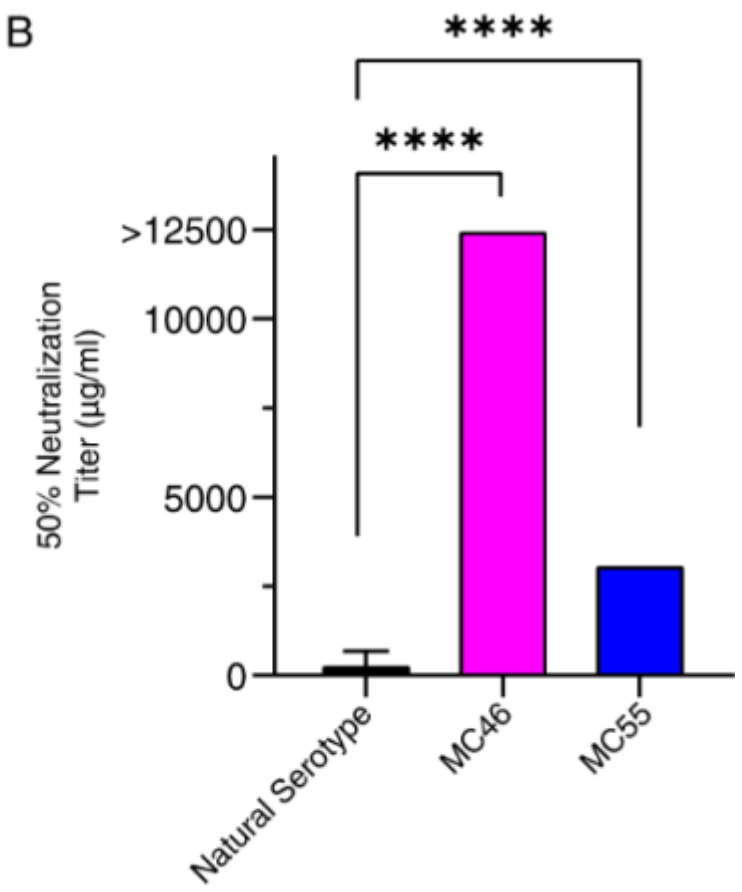

Figure S14. MC46 and MC55 capsids have significantly reduced neutralization by human antibodies both in vitro and in vivo.

(A) Values from SV40 DNA per 500ng of total DNA from individual mouse livers in an IVIG passive transfer experiment. AAV9, MC46, and MC55 with and without IVIG, and AAV9 with IVIG and IdeS. (B) Calculated 50% percent neutralization titers of AAV9, MC46, and MC55. Statistics calculated by one-way ANOVA, ****P≤0.0001.

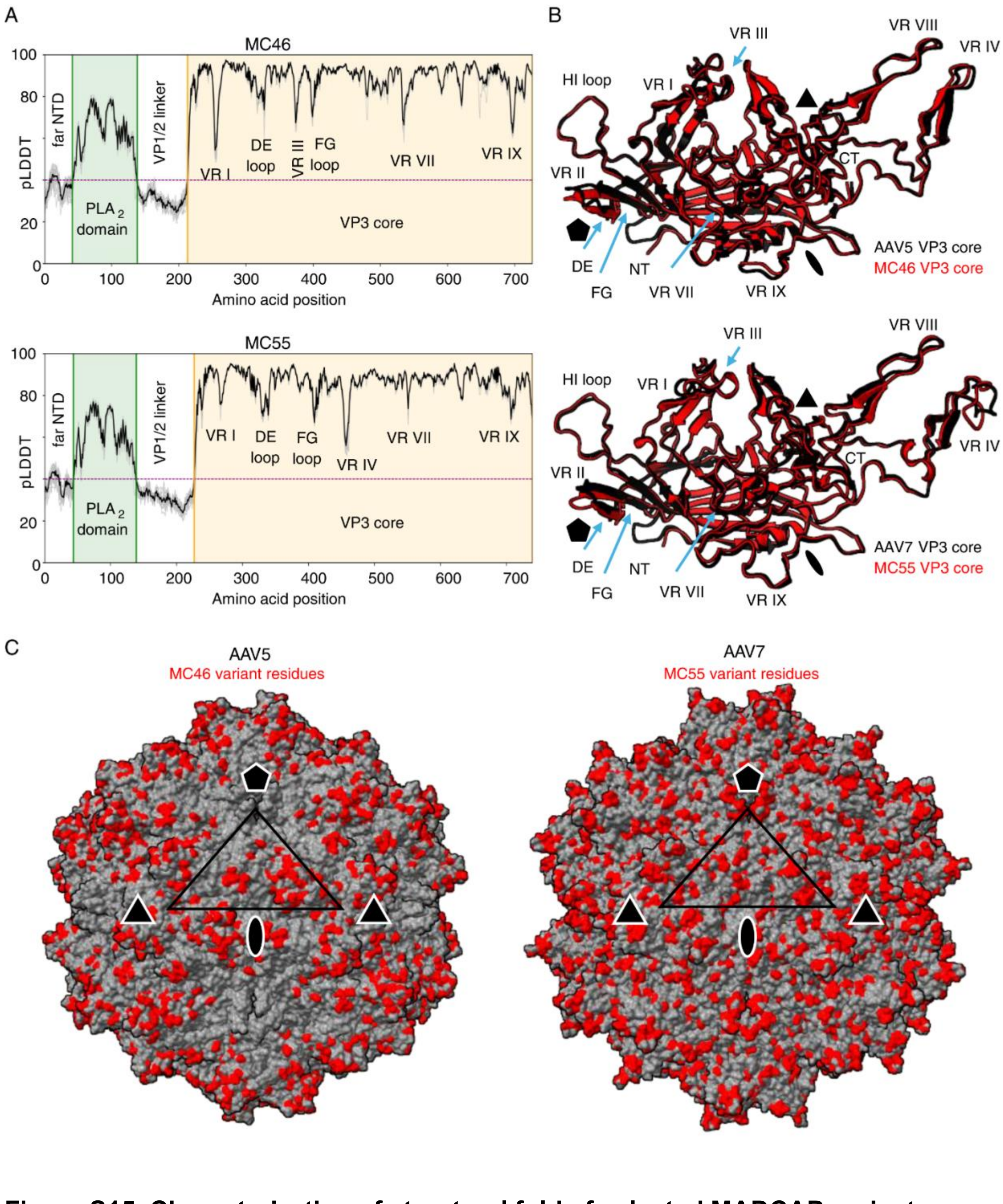

Figure S15. Characterization of structural fold of selected MADCAP variants.
One hundred thirty-nine MADCAPs were folded MC46 and MC55 are used as reference. (A) Plot of the pLDDT confidence metric of the 25 folded VP1 monomers (gray) and the average of all plots (black). A threshold of pLDDT=40 was used to denote position of the ordered PLA_2 domain (green region) and VP3 core (yellow region) from unordered regions of the capsid for analysis. Within each plot regions of interest and

low pLDDT are denoted. (B) Comparison of the VP3 core of MC46 to AAV5 (top) and MC55 to AAV7 (bottom). Regions previously noted in panel A as having systematically low pLDDT scores and with structural deviation from the compared virion are noted (arrows). (C) The 60mer capsid of AAV5 and AAV7 rendered in gray with the asymmetric unit denoted in an inset black triangle and symmetry features denoted. Residues that are variant from the known AAV5 and AAV7 structure are shown in red. Within this figure symmetry features of the monomer are denoted as 5-fold (pentagon), 3-fold (triangle), and 2-fold (oval).

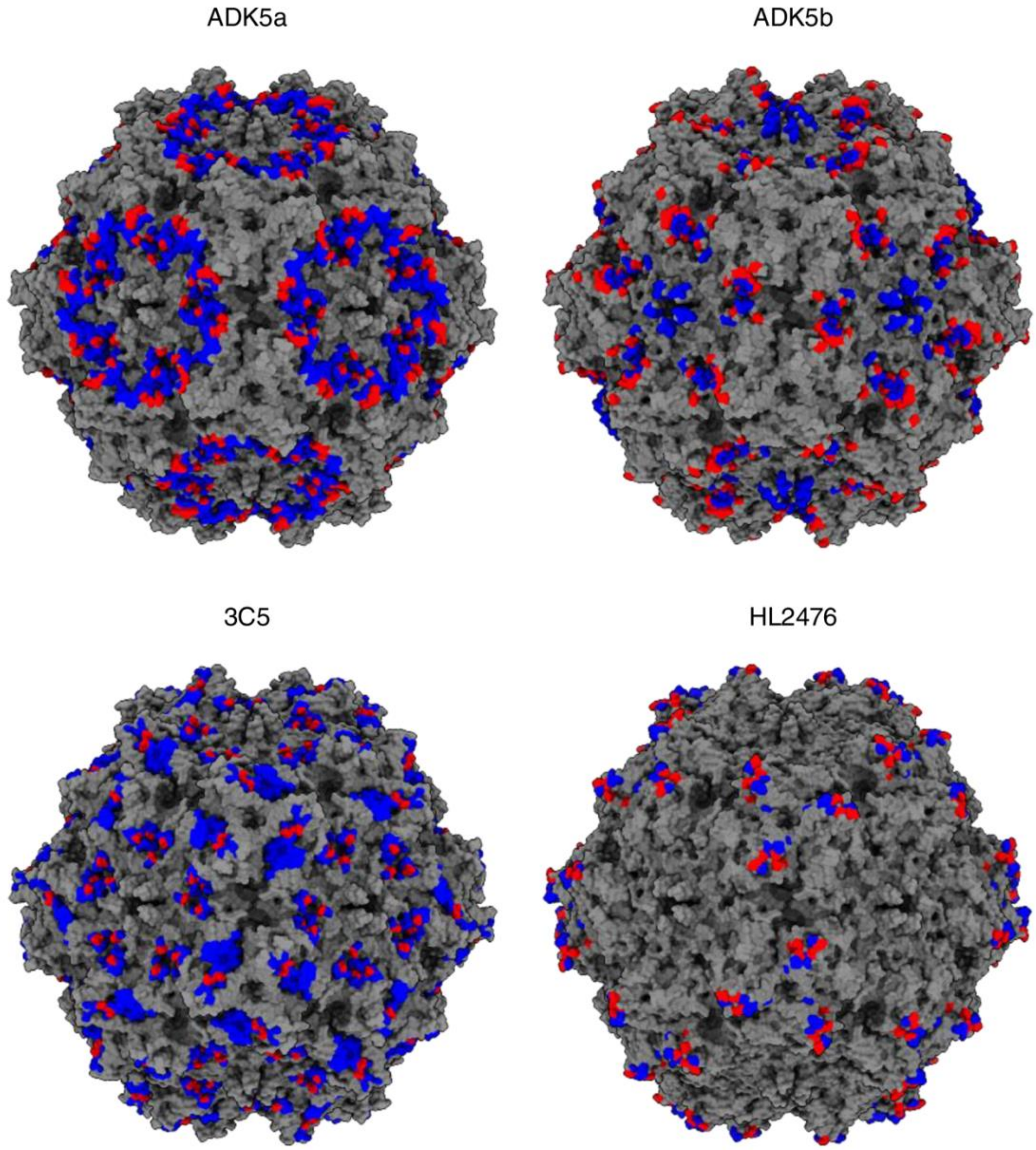

Figure S16. Point mutations on MC46 overlap the binding footprints in AAV5 for known antibodies. The AAV5 capsid (pdb 7KP3) is colored gray for the previous reported antibodies with the footprints of, ADK5b, 3C5, and HL2476 colored blue for positions that are shared between AAV5 and MC46 and red for those positions which differ.
