# Supplementary material for: MADCAP: isolation of novel nAb-naïve AAV capsids from metagenomic data
Source: J Virol. 2026 Sep 9;100(10):e00630-26. doi: 10.1128/jvi.00630-26 (PMC13712814; doi:10.1128/jvi.00630-26)
Supplement: Supplementary file 2 [file jvi.00630-26-s0004.pdf]

AAV	Cap Sequence Length	Documented Immunogenicity in AAV9 (HLA-B07:02)			Documented Immunogenicity in AAV9 (CD4) (HLA-A02:01, HLA-A02:05)			Documented Immunogenicity in AAV6/AAV9 (HLA-B07:02)			Documented Immunogenicity in AAV2/AAV6/AAV9 (HLA-B15:01)			Documented Immunogenicity in AAV9 (HLA-A02:01)			Documented Immunogenicity in AAV2 (HLA-B35:01)		
		Peptide 1	Position	HLA-Bining Score	Peptide 2	Position	HLA-Binding Score	Peptide 3	Position	HLA-Binding Score	Peptide 4	Position	HLA-Binding Score	Peptide 5	Position	HLA-Binding Score	Peptide 6	Position	HLA-Binding Score
AAV2	735	QPARKRLNF	165-173	0.880	KLFNIQVKEV	314-323	0.794	VPQYGYLTL	372-380	0.919	SQAVGRSSF	384-392	0.975	LIDQYLYYL	437-445	0.561	FPQSGVLIF	534-542	0.993
AAV6	736	QPAKKRLNF	165-173	0.919	KLFNIQVKEV	315-324	0.794	IPQYGYLTL	373-381	0.907	SQAVGRSSF	385-393	0.975	LIDQYLYYL	438-446	0.561	FPMSGVMIF	535-543	0.995
AAV9	736	QPAKKRLNF	165-173	0.919	KLFNIQVKEV	316-325	0.794	IPQYGYLTL	374-382	0.907	SQAVGRSSF	386-394	0.975	LIDQYLYYL	439-447	0.561	FPLSGSLIF	535-543	0.993
MC44	734	QPARKRLNF	164-172	0.880	KIFNIQVKEV	314-323	0.402	LPQYGYATL	372-380	0.882	NRPTERSSF	386-394	0.004	LVDQYLYRF	439-447	0.021	YALENTMIF	532-540	0.932
MC45	734	QPARKRLNF	164-172	0.880	KIFNIQVKEV	314-323	0.402	LPQYGYATL	372-380	0.882	NRPTERSSF	386-394	0.004	LVDQYLYRF	439-447	0.021	YALENTMIF	532-540	0.932
MC51	738	ETPAESLDI	165-173	0.000	KIFNIQVKEV	318-327	0.402	LPQYAYATL	376-384	0.818	PATDGRSAF	390-398	0.009	LIDQYLYRM	443-451	0.114	IAWDNTMVF	535-543	0.937
MC55	738	QTPAKKRPQG	161-171	0.006	KIFNIQVKEV	317-326	0.402	IPQYGYLTL	375-383	0.907	SQSVGRSSF	387-395	0.958	LVDQYLYYL	440-448	0.497	FPQHGVLVF	539-547	0.995
MC59	733	QPARKRLNF	162-170	0.880	KLFNIQVKEV	312-321	0.794	LPQYGYLTL	370-378	0.889	ALSLPQSAF	389-397	0.870	LIDQYLWYL	442-450	0.556	FTNGNELAF	528-536	0.771
MC60	733	QPARKRLNF	162-170	0.880	KLFNIQVKEV	312-321	0.794	LPQYGYLTL	370-378	0.889	ALSLPQSAF	389-397	0.870	LIDQYLWYL	442-450	0.556	FTNGNELAF	528-536	0.771
MC61	733	QPARKRLNF	162-170	0.880	KLFNIQVKEV	312-321	0.794	LPQYGYLTL	370-378	0.889	ALSLPQSAF	389-397	0.870	LIDQYLWYL	442-450	0.556	FTNGNELAF	528-536	0.771
MC62	733	QPARKRLNF	162-170	0.880	KLFNIQVKEV	312-321	0.794	LPQYGYLTL	370-378	0.889	ALSLPQSAF	389-397	0.870	LIDQYLWYL	442-450	0.556	FTNGNEMVF	528-536	0.643
MC63	733	QPARKRLNF	162-170	0.880	KLFNIQVKEV	312-321	0.794	LPQYGYLTL	370-378	0.889	ALSLPQSAF	389-397	0.870	LIDQYLWYL	442-450	0.556	FTNGNEMVF	528-536	0.643
MC64	733	QPARKRLNF	162-170	0.880	KLFNIQVKEV	312-321	0.794	LPQYGYLTL	370-378	0.889	ALSLPQSAF	389-397	0.870	LIDQYLWYL	442-450	0.556	FTNGNEMVF	528-536	0.643
MC65	733	QPARKRLNF	162-170	0.880	KLFNIQVKEV	312-321	0.794	LPQYGYLTL	370-378	0.889	ALSLPQSAF	389-397	0.870	LIDQYLWYL	442-450	0.556	FTNGNEMVF	528-536	0.643
MC66	729	QRLQGGAQA	171-179	0.001	KLFNIQVKEV	312-321	0.794	VPQYGYCTL	370-378	0.764	TQTMGKSSF	382-390	0.817	LMDQYLWYL	435-443	0.801	FPLSGDLVF	529-537	0.993
MC67	727	QPARKRLNF	164-172	0.880	KLFNIQVKEV	312-321	0.794	LPQYGYCTL	370-378	0.678	NDPVAQSSF	384-392	0.005	LIDQYLWYL	437-445	0.556	FQADGMIFA	525-533	0.002
MC46	725	SSDAEAGPS	165-173	0.000	KIFNIQVKEV	305-314	0.402	LPQYGYATL	363-371	0.882	DSPTERSSF	377-385	0.024	LVDQYLYRF	430-438	0.021	YALENTMIF	523-531	0.932
MC47	734	TPAKRPRPL	168-176	0.990	KIFNIQVKEV	313-322	0.402	LPQYAYATL	371-379	0.818	PATNGRSAF	385-393	0.006	LLDQYLYKH	438-446	0.011	ISWDNTMVF	531-539	0.645
MC48	739	TPAKRPRPL	173-181	0.990	KIFNIQVKEV	318-327	0.402	LPQYAYATL	376-384	0.818	PATNGRSAF	385-393	0.006	LDQYLYKHT	444-452	0.000	ISWDNTMVF	536-544	0.645
MC49	734	KPSSSGSVN	162-170	0.027	KVFNIQVKEV	310-319	0.327	LPQYGYATL	368-376	0.882	SRSSFFCLE	390-398	0.000	LVDQYLYRA	439-447	0.103	IAVDNTLIF	532-540	0.859
MC50	734	QPSRKRLNF	164-172	0.832	KIFNIQVKEV	313-322	0.402	LPQYGYATL	371-379	0.882	NHPTERSSF	387-395	0.005	LVDSYLYRF	440-448	0.027	IALENSLIF	533-541	0.740
MC52	694	QPASSLGAD	153-161	0.002	KIFNIQVKEV	275-284	0.402	LPQYGYATL	333-341	0.882	ENPTERSSF	347-355	0.006	LVDQYLYRF	400-408	0.021	YALENTMIF	492-500	0.932
MC69	721	QPARKRLNF	161-169	0.880	KLFNIQVKEV	308-317	0.794	LPQYGYLTL	366-374	0.889	NQALPQSSF	378-386	0.897	LIDQYLWYL	431-439	0.556	ITADGQLIF	517-525	0.335
MC72	715	QDKRPNLDV	150-158	0.001	KLFNIQVKEV	300-309	0.794	LPQYGYCTR	358-366	0.005	SNPTPRSAF	372-380	0.063	LIDQYLYYL	425-433	0.561	KLDGNGLIF	510-518	0.055
MC73	715	QDKRPNLDV	150-158	0.001	KLFNIQVKEV	300-309	0.794	LPQYGYCTR	358-366	0.005	SNPTPRSAF	372-380	0.063	LIDQYLYYL	425-433	0.561	KLDGNGLIF	510-518	0.055
MC74	743	QTPKKNKKP	167-175	0.000	RLFNIQVKEV	321-330	0.713	IPQYGYCTL	379-387	0.731	NEAVDRSAF	391-399	0.049	LVDQYLWAF	444-452	0.011	QAMQNTLAF	541-549	0.957
MC75	742	QTPKKNKKP	167-175	0.000	RLFNIQVKEV	320-329	0.713	IPQYGYCTL	378-386	0.731	NEAVDRSAF	390-398	0.049	LVDQYLWAF	443-451	0.011	QAMQNTLAF	540-548	0.957
MC76	742	QTPKKNKKQ	167-175	0.000	RLFNIQVKEV	320-329	0.713	IPQYGYCTL	378-386	0.731	NEAVDRSAF	390-398	0.049	LVDQYLWAF	443-451	0.011	QAMQNTLAF	540-548	0.957
MC77	742	QTPKKNKKP	167-175	0.000	RLFNIQVKEV	320-329	0.713	IPQYGYCTL	378-386	0.731	NEAVDRSAF	390-398	0.049	LVDQYLWAF	443-451	0.011	QAMQNTLAF	540-548	0.957
MC78	717	QDKRPNLDV	152-160	0.001	KLFNIQVKEV	302-311	0.794	LPQYGYCTR	360-368	0.005	SNPTPRSAF	374-382	0.063	LIDQYLYYL	427-435	0.561	KLDGNGLIF	512-520	0.055
MC79	715	QDKRPNLDV	152-160	0.001	KLFNIQVKEV	301-310	0.794	LPQYGYCTR	359-367	0.005	SNPTPRSAF	373-381	0.063	LIDQYLYYL	426-434	0.561	KFDGNGLIF	511-519	0.093
MC81	715	QDKRPNLDV	152-160	0.001	KLFNIQVKEV	301-310	0.794	LPQYGYCTR	359-367	0.005	SNPTPRSAF	373-381	0.063	LIDQYLYYL	426-434	0.561	KFDGNGLIF	511-519	0.093
MC82	715	QDKRPNLDV	152-160	0.001	KLFNIQVKEV	301-310	0.794	LPQYGYCTR	359-367	0.005	SNPTPRSAF	373-381	0.063	LIDQYLYYL	426-434	0.561	KFDGNGLIF	511-519	0.093
MC84	737	QGKAPAQTG	172-180	0.001	RLFNIQVKEV	315-324	0.713	IPQYGYCTL	373-381	0.731	NEAVDRSAF	385-393	0.049	LVDQYLWAF	438-446	0.011	QAMQNTLAF	535-543	0.957
MC85	727	QPSRRRLDF	157-163	0.720	KLFNIQVKEV	307-316	0.794	LPQYGYATL	365-373	0.882	GNPTERSSF	379-387	0.019	LVDQYLYRF	432-440	0.021	ICLDNSLIF	525-533	0.100
MC86	727	QPSRRRLDF	157-163	0.720	KLFNIQVKEV	307-316	0.794	LPQYGYATL	365-373	0.882	GNPTERSSF	379-387	0.019	LVDQYLYRF	432-440	0.021	ICLDNSLIF	525-533	0.100
MC88	727	QPSRRRLDF	157-163	0.720	KLFNIQVKEV	307-316	0.794	LPQYGYATL	365-373	0.882	GNPTERSSF	379-387	0.019	LVDQYLYRF	432-440	0.021	ICLDNSLIF	525-533	0.100
MC89	729	QSARKKLDF	156-162	0.002	KLFNIQVKEV	310-319	0.794	LPQYGYATL	368-376	0.882	GNPVERSAF	382-390	0.012	LVDQYLYRF	435-443	0.021	YALDNTLVF	527-535	0.979
MC90	731	QGASRRRLDF	158-167	0.001	KLFNIQVKEV	311-320	0.794	VPQYGYATL	369-377	0.911	GNPVERSAF	383-391	0.012	LVDQYLYRF	436-444	0.021	YALDATMVF	529-537	0.964
MC91	722	QDKRPNLDV	152-160	0.001	KLFNIQVKEV	303-312	0.794	LPQYGYATL	361-369	0.882	GDPTERSSF	375-383	0.006	LVDQYLYRF	428-436	0.021	VCLDNSLIF	520-528	0.086
MC92	722	QDKRPNLDV	152-160	0.001	KLFNIQVKEV	303-312	0.794	LPQYGYATL	361-369	0.882	GDPTERSSF	375-383	0.006	LVDQYLYRF	428-436	0.021	VCLDNSLIF	520-528	0.086
MC135	726	QPASSLGAD	183-191	0.002	KIFNIQVKEV	305-314	0.402	LPQYGYATL	363-371	0.882	DNPTERSSF	377-385	0.005	LVDQYLYRF	430-438	0.021	YALENTMIF	523-531	0.932
MC138	733	QPSRKRLNF	164-172	0.832	KIFNIQVKEV	311-320	0.402	LPQYGYATL	369-377	0.882	DRPTDRSSF	385-393	0.003	LVDSYLYRL	438 -446	0.481	IALENSVIF	531-539	0.736
MC139	743	QTPKKNKKP	167-175	0.000	RLFNIQVKEV	320-329	0.713	IPQYGYCTL	378-386	0.731	NEAVDRSAF	390-398	0.049	LVDQYLWAF	443 -451	0.011	QAMQNTLAF	540-548	0.957

Natural Serotypes

Neutralizable MADCAP

Non-neutralizable MADCAP

Table S3. ***In silico*** **predicted HLA-binding for peptides corresponding to 6 immunodominant epitopes from AAV2, AAV6, and AAV9.**

HLA-binding scores generated through the Immune Epitope Database (1=most stable binding to HLA-I complex; 0= least stable binding). Highlighted (yellow) peptides are naturally presented and have documented T-cell immunogenicity in natural serotypes.

Table S6. Tissue homogenization and nucleic extraction for individual tissue types.

Tissue type	Tissue disrupter tube	Buffer; homogenization cycle	Nucleic acid extraction kit
NHP brain: cortex (motor, frontal, cingulate, sensory, occipital, insular, temporal, entorhinal, parietal), hippocampus, thalamus, caudate, putamen, globus pallidus, subthalamic nucleus, sustantia nigra, cerebellar denate, medulla, cerebellum, midbrain/ pons	1.4 mm beads (Fisherbrand, 15555799)	Buffer RLT (Qiagen, 79216) with β-mercaptoethanol; 5 m/sec, 20 seconds, 1 cycle with Omni tissue homogenizer (Fisherbrand, 15340163)	Allprep DNA/ RNA/ protein kit (Qiagen, 80004)
NHP brain: medulla pyramid, corpus callosum, internal capsule, anterior commissure, centrum semiovale, cerebral peduncle, brachium pontis, cerebellum arbor vitae	2.8 mm beads (Fisherbrand, 15565799)	Buffer RLT (Qiagen, 79216) with β-mercaptoethanol; 5 m/sec, 20 seconds, 2 cycles with 10 second pause with Omni tissue homogenizer (Fisherbrand, 15340163)	Allprep DNA/ RNA/ protein kit (Qiagen, 80004)
NHP spinal cord	2.8 mm beads (Fisherbrand, 15565799)	Buffer RLT (Qiagen, 79216) with β-mercaptoethanol; 5 m/sec, 20 seconds, 2 cycles with 10 second pause with Omni tissue homogenizer (Fisherbrand, 15340163)	Allprep DNA/ RNA/ protein kit (Qiagen, 80004)
NHP dorsal root ganglion	1.4 mm beads (Fisherbrand, 15555799)	Buffer RLT (Qiagen, 79216) with β-mercaptoethanol; 5 m/sec, 20 seconds, 1 cycle with Omni tissue homogenizer (Fisherbrand, 15340163)	Allprep DNA/ RNA/ protein kit (Qiagen, 80004)
Mouse brain	5mL tube with 3/8" metal ball (Cole-Parmer, 2155)	Buffer RLT (Qiagen, 79216) with β-mercaptoethanol; 1100 m/sec, 10 seconds, 2 cycles with 30 second pause with HG-600 Geno/ Grinder (Cole-Parmer, 2010115)	Allprep DNA/ RNA/ protein kit (Qiagen, 80004)

Tissue type	Tissue disrupter tube	Buffer; homogenization cycle	Nucleic acid extraction kit
NHP and mouse liver	1.4 mm beads (Fisherbrand, 15555799)	Buffer RLT (Qiagen, 79216) with β-mercaptoethanol; 5 m/sec, 20 seconds, 1 cycle with Omni tissue homogenizer (Fisherbrand, 15340163)	Allprep DNA/ RNA/ protein kit (Qiagen, 80004)
NHP and mouse spleen	2.8 mm beads (Fisherbrand, 15565799)	Buffer RLT (Qiagen, 79216) with β-mercaptoethanol; 5 m/sec, 20 seconds, 2 cycles with 10 second pause with Omni tissue homogenizer (Fisherbrand, 15340163)	Allprep DNA/ RNA/ protein kit (Qiagen, 80004)
NHP and mouse kidney	2.8 mm beads (Fisherbrand, 15565799)	Buffer RLT (Qiagen, 79216) with β-mercaptoethanol; 5 m/sec, 20 seconds, 2 cycles with 10 second pause with Omni tissue homogenizer (Fisherbrand, 15340163)	Allprep DNA/ RNA/ protein kit (Qiagen, 80004)
NHP and mouse muscle:	5mL tube (Fisher Scientific, 2241-PEF-200) with 3/8" metal ball (Cole-Parmer, 2155)	QuantiGene homogenization buffer (Thermofisher, QG0517) with Proteinase K (Qiagen, 19133); 1300 m/sec, 10 seconds, 2 cycles with 30 second pause with HG-600 Geno/ Grinder (Cole-Parmer, 2010115)	MiRNeasy Mini kit (Qiagen, 217004)
NHP and mouse skin	5mL tube (Fisher Scientific, 2241-PEF-200) with 3/8" metal ball (Cole-Parmer, 2155)	QuantiGene homogenization buffer (Thermofisher, QG0517) with Proteinase K (Qiagen, 19133); 1300 m/sec, 10 seconds, 2 cycles with 30 second pause with HG-600 Geno/ Grinder (Cole-Parmer, 2010115)	MiRNeasy Mini kit (Qiagen, 217004)

Table S7. Primer sets for amplification of barcodes in viral pools or in vivo.

Name	Sequence
OL_777 (forward)	5'-ACACTCTTTCCCTACACGACGCTCTTCCGATCTAAATGCTTCTTTCCAGTTTT-3'
OL_778 (reverse)	5'-GACTGGAGTTCAGACGTGTGCTCTTCCGATCTTGAGGTGGAAGGTTGGGACTT-3'
OL_779 (forward)	5'-ACACTCTTTCCCTACACGACGCTCTTCCGATCTTGCTTCTTTCCAGTTTTCAG-3'
OL_780 (reverse)	5'-GACTGGAGTTCAGACGTGTGCTCTTCCGATCTAGTGAGGTGGAAGGTTGG-3'
OL_1094 (forward)	5'-ACACTCTTTCCCTACACGACGCTCTTCCGATCTNNNNACCTAGAAATGCTTCTTTCC-3'
OL_1095 (reverse)	5'-GACTGGAGTTCAGACGTGTGCTCTTCCGATCTNNNNGAAGGTTGGGACTTTTATCA-3'

Table S8. Experimental design for mouse IVIG passive transfer study

	Group	AAV Treatment<br>I.V Tail vein (100µl)<br>Day 0	n	IVIG<br>I.V Tail vein (180µl)<br>Day -1	Immune Modulation<br>Day -1<br>(30 mins post IVIG)
1	Control	-	5	-	-
2	MC #46	MC#46<br>1xE11 VG per mouse	5	-	-
3	MC #46 + IVIG		5	IVIG 9mg/mouse	-
4	MC#55	MC#55<br>1xE11 VG per mouse	5	-	-
5	MC#55 + IVIG		5	IVIG 9mg/mouse	-
6	rAAV9	rAAV9<br>1xE11 VG per mouse	5	-	-
7	rAAV9+IVIG		5	IVIG 9mg/mouse	-
8	rAAV9 + IVIG + IdeS		5	IVIG 9mg/mouse	IdeS<br>25µg/mouse
