# Supplementary material for: MADCAP: isolation of novel nAb-naïve AAV capsids from metagenomic data
Source: J Virol. 2026 Sep 9;100(10):e00630-26. doi: 10.1128/jvi.00630-26 (PMC13712814; doi:10.1128/jvi.00630-26)

Supplementary File 1. Full Phylogenetic treee of capsids used in the study.

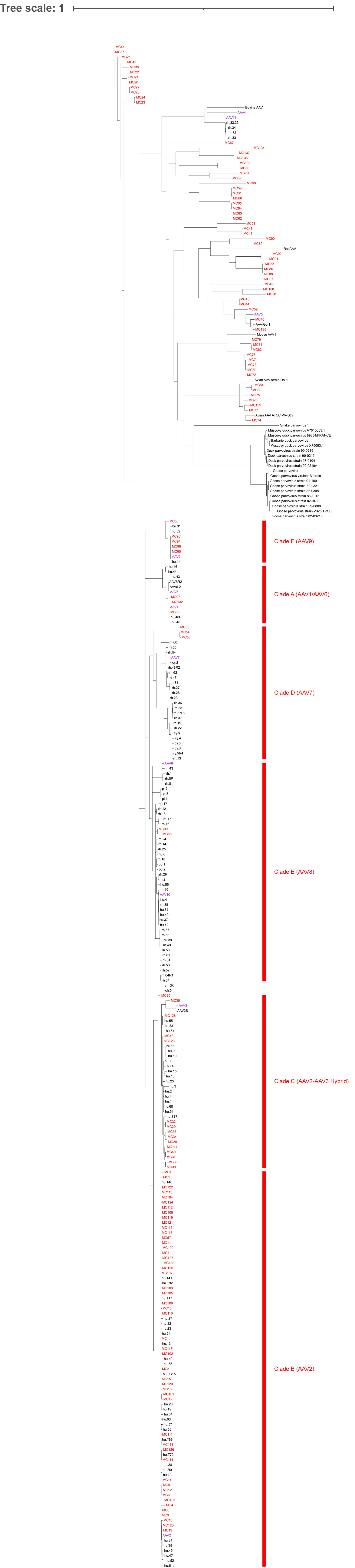

Supplement: Supplementary file 3 [file jvi.00630-26-s0007.pdf]
